# Enhancing Optoelectronic Anisotropy in Highly Oriented Thin Films by Fluorine Substitution in Novel Semiconducting Polymers

**DOI:** 10.1021/acsami.4c08566

**Published:** 2024-09-17

**Authors:** Shubham Sharma, Moulika Desu, Guan-Lin Chen, Kai-Wei Tseng, Kumar Vivek Gaurav, Zhe-Yu Liu, Kuang-Hao Cheng, Safalmani Pradhan, Palraj Ranganathan, Pang-Hsiao Liu, Xiang-Ling Chiu, Hirofumi Tanaka, Jyh-Chien Chen, Chin-Ti Chen, Chi-An Dai, Leeyih Wang, Shyam S. Pandey

**Affiliations:** † Graduate School of Life Science and Systems Engineering, Kyushu Institute of Technology, 2-4 Hibikino, Wakamatsu, Kitakyushu, Fukuoka 808-0196, Japan; ‡ Center for Condensed Matter Science, National Taiwan University, Taipei 10617, Taiwan; § Center of Atomic Initiative for New Materials, National Taiwan University, Taipei 10617, Taiwan; ⊥ Institute of Polymer Science and Engineering, National Taiwan University, Taipei 10617, Taiwan; ¶ Department of Chemical Engineering, National Taiwan University, Taipei 10617, Taiwan; □ Department of Materials Science and Engineering, National Taiwan University of Science and Technology, Taipei, 10607, Taiwan; ■ Department of Brain Science, Kyushu Institute of Technology, 2-4 Hibikino, Wakamatsu, Kitakyushu, Fukuoka 808-0196, Japan; ○ Institute of Chemistry, Academia Sinica, Taipei 11529, Taiwan

**Keywords:** Semiconducting polymers, Thin films, Device Fabrication, Planarity, Organic field transistors, Fluorination

## Abstract

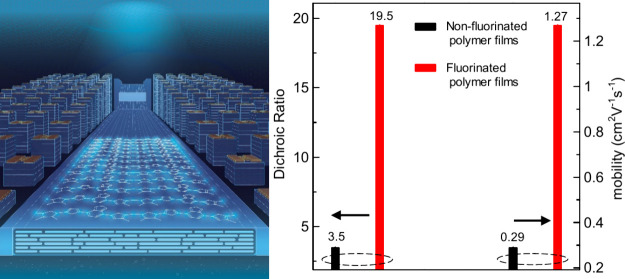

The recent past has witnessed remarkable progress in organic electronics, driven by the quest for flexible, lightweight, and cost-effective electronic devices. Semiconducting polymers (SCPs) have emerged as key materials in this field, offering unique electronic and optoelectronic properties along with mechanical flexibility. This study focuses on designing, synthesizing, and utilizing novel donor–acceptor (D–A) copolymer-based SCPs introducing a difluorothiophene moiety in the polymeric backbone. The importance of fluorine substitution for backbone planarity was verified by density functional theory calculations, comparing it with a nonfluorine substituted counterpart. Through the Unidirectional Floating Film Transfer Method (UFTM), we fabricated highly oriented thin films, resulting in increased optical anisotropy with dichroic ratios reaching 19.3 in PC20-FT thin films, one of the highest optical anisotropy observed for solution processable SCP thin films. X-ray diffraction and atomic force microscopy results validated the increase in the crystallinity and domain size with the increasing alkyl chain length. Finally, we elucidate these findings in the context of electrical applications by fabricating organic field-effect transistors revealing anisotropic charge transport achieving a promising mobility of 1.24 cm^2^V^–1^s^–1^ and mobility anisotropy of 39.5. This study offers insights into the design principles and performance optimization of SCP-based devices, paving the way for advancements in plastic electronics.

## 1 Introduction

Over the past few decades, organic electronics have witnessed significant advancements, fueled by the pursuit of flexible, lightweight and cost-effective electronic devices.^1−4^ Semiconducting polymers (SCPs), an intriguing class of materials, have emerged as vital in this paradigm shift. Integrating the intrinsic features of polymers with semiconducting characteristics has paved the path for various applications, from field-effect transistors (FETs) and organic photovoltaics to sensors and flexible displays.^5−8^ These materials have unique electronic and optoelectronic properties, and mechanical flexibility, which can be tailored through subtle molecular design.^9^ The quest for the search of novel materials, which involves unique structures, properties, and functionalities, goes hand in hand with engineering the materials into various optoelectronic devices, thus unlocking unprecedented device performance and functionality.^10−12^ Despite the remarkable progress witnessed in the last two decades, FET performances of the SCP-based materials have been constrained. The limitation, while stemming from the inherent structure of the SCPs as well as intricate nature of polymer chains, has widely been flexible and often disordered configurations, introducing energetic disorders, which obstructs the efficient charge transport.^13,14^ Furthermore, the SCP film with short-range π-orbital overlap and intricate intermolecular interactions hampers field-effect mobilities of polymer FETs.^15^ In recent years, there has been notable progress in the development of low-bandgap SCPs of the donor–acceptor (D–A) architecture, exhibiting charge carrier mobilities that outperform those observed for amorphous silicon. With the presence of electron-rich (donor) and electron-deficient (acceptor) moieties with the same polymer backbone, these SCPs engender strong π–π as well as donor–acceptor interactions, facilitating fast interchain carrier hopping.^16,17^

Furthermore, their bandgap is effectively reduced by the interchain D–A interactions, rendering them highly suitable materials for applications in FETs and solar cells. Notably, the judicious selection of donor and acceptor moieties, in conjugation with a comprehensive understanding of structure–property relationships, have resulted in excellent breakthroughs in FET performances.^18,19^ To further enhance the charge carrier transport, the molecular structure of these low band gap polymers needs meticulous design considering their close relation with the charge transport parameters, such as field-effect mobility, on–off ratio, etc. Recently, the introduction of Fluorine (F) atoms into polymer chains has led to the creation of remarkably efficient charge transport materials.^20−22^ Fluorine possesses a compact van der Waals radius and stands as the most electronegative element, with a Pauling electronegativity of 4.0. Incorporating F on an electron rich unit of a D–A system can offer an intriguing strategy to modify the energy levels of the molecular orbital in the system. It can effectively lower the highest occupied molecular orbital (HOMO) energy level of the donor unit, making it easy for the polymer to donate electrons, thus enhance the charge carrier transport in FETs.^23^ Fluorine substitution has a high impact on the intra- and intermolecular interactions, thereby playing a pivotal role in the solid-state polymer arrangement with cofacial π–π stacking.^24−26^ Furthermore, past reports indicate that noncovalent attractive forces operating among adjacent moieties through intra/intermolecular hydrogen bonding or dipole–dipole interactions have contributed to maintaining the polymer chains in a planar conformation, while simultaneously preserving their solubility. Guo et al. also reported an intramolecular S (thiazolyl)···O (alkoxy) interactions which helped in an extensive π–conjugation, lowering the band gap, and planarizing the conformation of their SCP. Further, they reported an enhanced hole mobility of polymer FETs.^27^ Takacs et al. recently examined the correlations of the dipole–dipole interaction and the conformational stability to the self-assembly of the SCPs.^28^ Sun et al. recently reported an n-type SCP, wherein an intramolecular S···F noncovalent interaction enhanced the backbone planarity attributed to high electronegativity of fluorine.^29^ Across all of the aforementioned reports, a reduced distortion in the backbone planarity of the SCPs has consistently led to enhanced device performance.

**Figure 1:**
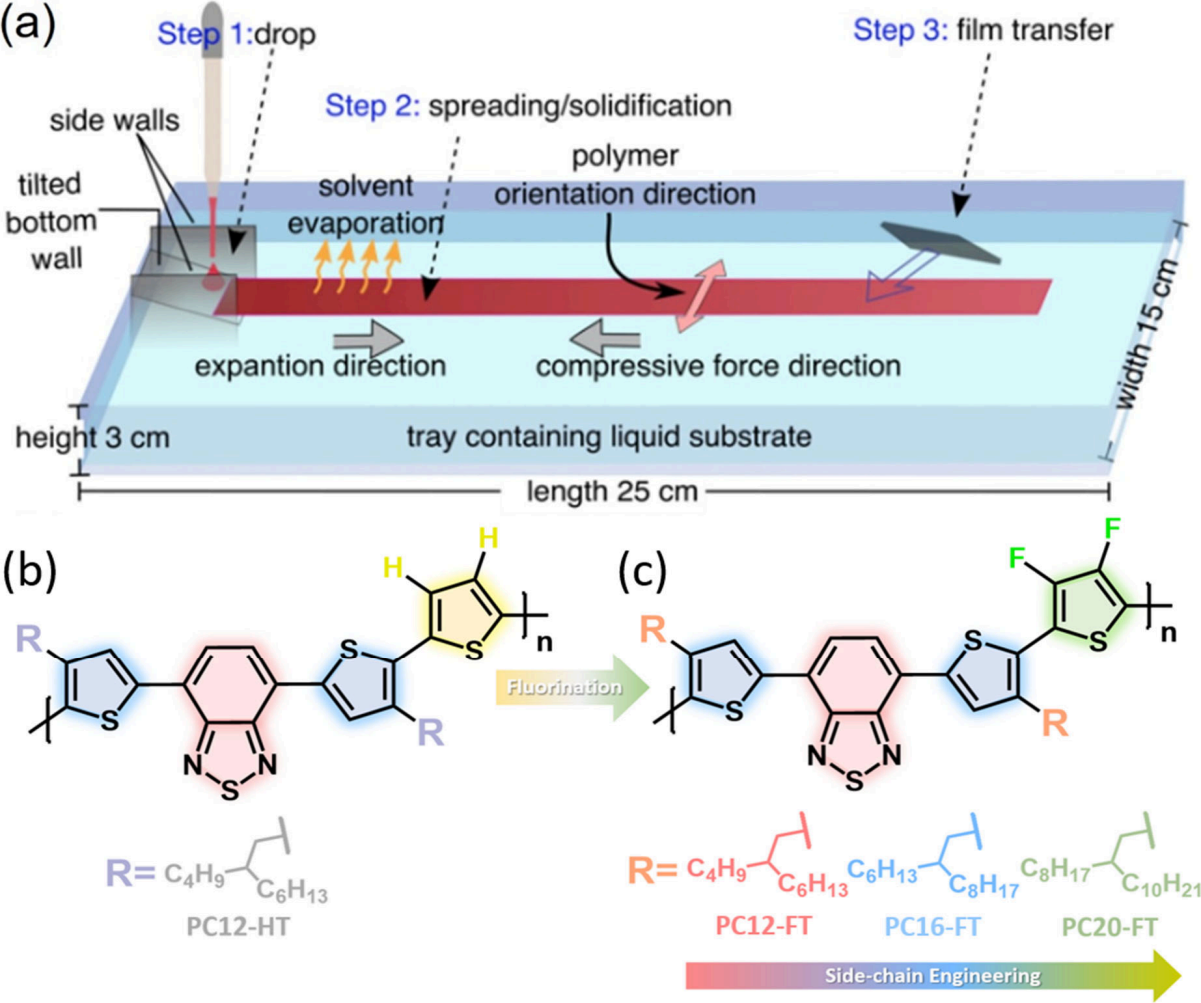
(a) Schematic representation of the UFTM, and chemical structure of solution processable organic semiconductors (b) PC12-HT, and (c) PC-FT.

The introduction of a high-mobility thermionic liquid crystalline polymer with crystalline morphologies offers a solution to attain effective ordering and packing of organic materials apart from mitigating the defects formed at the D–A interface. Liquid crystals exhibit ordering as well as mobility across molecular and macroscopic scale because of their nature as dynamic self-assembled functional soft materials.^30−32^ Orientation of these SCPs and their control in their thin films is of paramount importance to control the device performance owing to the inherent tendency of the SCPs toward molecular self-assembly. At the molecular level, the degree of alignment of crystalline domains governs the charge transport behavior. There are numerous methods for the orientation of SCP domains in thin films. However, conventional thin film fabrication methods lack control over film morphology, precise alignment of molecular domains, material wastage, or usage of expensive instruments.^33,34^ Recently, Yen-Han et. al proposed novel DA type SCPs with fluorinated backbones have been processed using spin-coating method and FETs were fabricated, but these films lacked orientation, due to which a maximum hole mobility of ∼0.1 cm^2^V^–1^s^–1^ was reported.^35^ We have recently developed and improvised the Unidirectional Floating Film Transfer Method (UFTM) for the fabrication of highly oriented and large area thin films of SCPs (Figure 1a).^36−38^ In this method, a drop SCP ink is placed on the orthogonal liquid substrate (subphase liquid) at an optimum temperature and concentration and viscosity. Due to the difference in surface tensions of the two liquids, the SCP ink spreads over the subphase liquid, and with simultaneous evaporation of the SCP ink solvent, a large area, thin solid film is formed floating over the subphase liquid. As elaborated in our previous reports, the thin film is oriented perpendicular to the flow direction of the SCP ink, due to hydraulic resistance provided by the viscous subphase liquid.^38,39^

In this work, we report on the synthesis and characterization of novel solution processable SCPs utilizing benzothiadiazole, featuring difluorothiophene substitution in its backbone. First, we have compared the SCP, namely Poly[(3-(2-butyloctyl)-3′,4′-difluoro-5′-methyl-[2,2′-bithiophen]-5-yl)-7-(4-(2-butyloctyl)-5-methylthiophen-2-yl)benzo[c][1,2,5] thiadiazole] abbreviated as PC12-HT (Figure 1b) with its fluorinated substitution namely Poly[(3-(2-alkyl)-3′,4′-difluoro-5′-methyl-[2,2′-bithiophen]-5-yl)-7-(4-(2-butyloctyl)-5-methylthiophen-2-yl)benzo[c][1,2,5] thiadiazole], abbreviated as PC-FT (Figure 1c), thereby exhibiting the effect of fluorine substitution in increasing the intra- and intermolecular interactions and planarizing. Subsequently, we have demonstrated how varying alkyl chain lengths contribute to the backbone alignment and chain packing of PC-FT, for which we have taken three variations of alkyl chains into consideration, PC12-FT, PC16-FT, and PC20-FT. We have utilized UFTM as a mode of highly oriented thin film fabrication. Finally, we have fabricated FETs using these SCPs and achieved a hole mobility of >1 cm^2^V^–1^s^–1^.

## 2 Results

### 2.1 Molecular Design, Synthesis, And Characterization

**Scheme 1:**
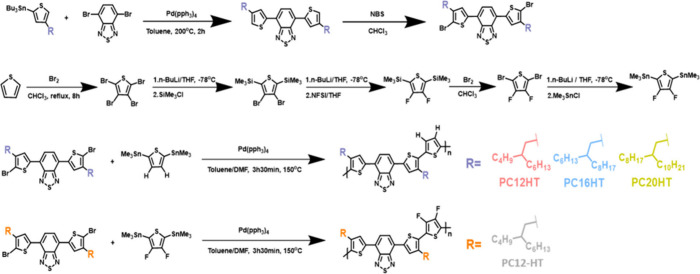
Synthesis of Monomers and Polymers PC12-HT, PC12-FT, PC16-FT, and PC20-FT

The synthetic pathways for four newly designed solution processable DA copolymers PC12HT, PC12-FT, PC16-FT, and PC20-FT with chemical structures as shown in Figure 1 are delineated in Scheme 1. The structures of monomers M1 and M2 are further confirmed through ^1^H, ^19^F and ^13^C NMR analysis. PC12-HT, PC12-FT, PC16-FT, and PC20-FT polymers are synthesized via microwave-assisted Stille coupling polymerization, utilizing dibromo monomer M1 along with either 3,4-difluoro-2,5-bis(trimethylstannyl)thiophene M2 or 2,5-bis(trimethylstannyl)thiophene M3, with Pd(PPh_3_)_4_ serving as the catalyst as reported previously.^35^ Comprehensive details regarding the synthesis of these SCPs have been provided in the Supporting Information. The weight-average molecular weight (*M*_w_) was measured to be 25.2 kDa (polydispersity index, PDI = 1.61), 15.8 kDa (1.55), 30.1 kDa (1.45), and 22.9 kDa (1.82) for PC12-HT, PC-12FT, PC16-FT, and PC20-FT, respectively.

A promising approach for designing high-performance SCP involves the incorporation of a planar backbone consisting of alternating D and A units. Planar backbone allows swift intrachain charge transfer as well as strong interchain interactions leading to facile interchain charge hopping. Benzothiadiazole is a commonly utilized electron-deficient unit due to its ability to facilitate intra- and intermolecular interactions, leading to enhanced intramolecular charge transfer and charge carrier mobility within the SCP.^40,41^ Maintaining a coplanar conformation of the conjugated backbone is critical for achieving high crystallinity and hole mobility, as this arrangement extends the π-conjugation and promotes favorable π–π stacking between adjacent SCP chains.^42,43^ Incorporating fluorine atoms into the polymer backbone is an effective strategy to improve coplanarity. The high electronegativity of fluorine enables the formation of stabilizing F···S halogen bonding through intramolecular Coulombic interactions with adjacent thiophene sulfur atoms, known as nonbonding interaction.^44,45^ This encourages organized molecular packing and enhanced crystallinity. Additionally, the presence of alkyl side chains is essential for promoting a highly ordered orientation of SCP chains and ensuring the SCPs’ solubility in organic solvents during solution processes.

**Figure 2:**
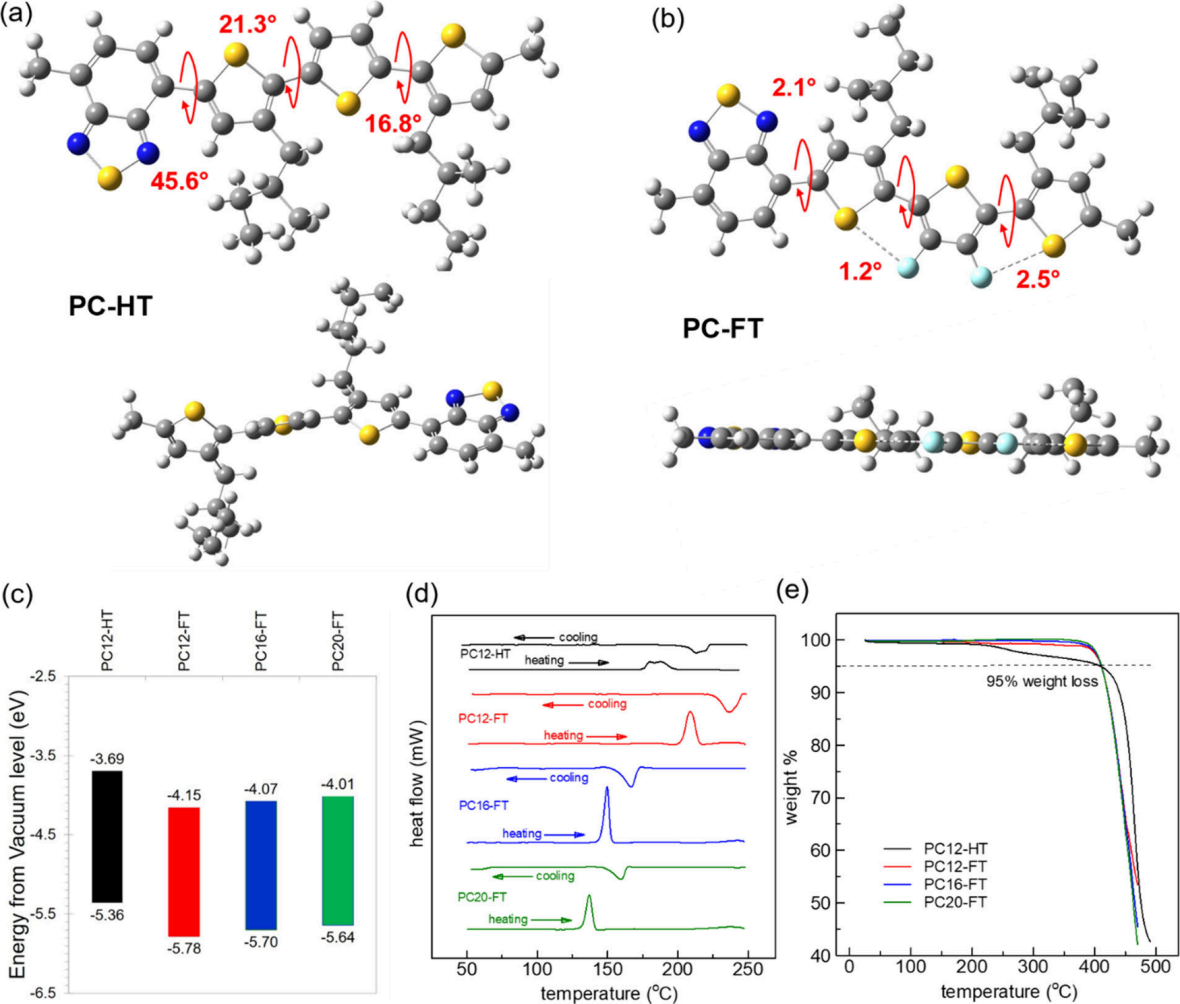
Electronic structure of (a) PC-HT, and (b) PC-FT, showing structural planarity using density functional theory. (c) HOMO and LUMO energy levels, (d) differential scanning calorimetry, and (e) thermogravimetric analysis of PC12-HT, PC12-FT, PC16-FT and PC20-FT.

Computational studies employing density functional theory (DFT) with the B3LYP hybrid functional and 6-311G(d,p) basis set were performed to investigate the molecular geometries and properties of two types of SCPs, PC-HT and PC-FT, as depicted in Figure 2**a,b**. To simplify the calculations and obtain more accurate results, monomer units with relatively shorter and branched alkyl chains (2-methyl butyl) instead of long alkyl chains were considered for easiness and small computation cost. Notably, to elucidate the F···S nonbonding interaction, a dative bond was included during the DFT calculations. The dihedral angle between the different moieties was estimated to be 45.6°, 21.3°, and 16.8° for PC-HT and significantly smaller 2.1°, 1.2°, and 2.5° for PC-FT. The dihedral angles involving other bonds were similar and minimal for both SCPs, indicating that PC-FT exhibits a much more planar conformation compared to its PC-HT counterpart, as illustrated in Figure 2(b). Later, binding energies were calculated by considering cofacial dimer configurations, as shown in Supporting Figure S1. The head-to-tail (HT) configuration was found to be the most stable among the possible configurations due to the minimization of electrostatic repulsion between the monomers and favorable dispersive interactions. The calculated binding energies of the HT-type cofacial dimers were found to be −1.06 kcal mol^–1^ and −2.56 kcal mol^–1^ for PC-HT and PC-FT, respectively. The higher binding energy value for PC-FT indicates a more stable cofacial dimer arrangement with their energies clearly stabilized upon fluorine substitution. This stabilization effect can be attributed to the enhanced dispersive interactions and electrostatic complementarity arising from the fluorine substituents. Furthermore, we calculated the torsional potential energy profiles for PC-HT and PC-FT by rotating the two thiophene moieties and difluoro-thiophene and thiophene moieties, respectively about the inter-ring bond (Supporting Figure S2). For PC-HT, the minimum energy conformation corresponds to a dihedral angle of 340° (−20°), while for PC-FT, the minima are located at 0° and 360°. Additionally, the torsional curve for PC-FT exhibits symmetric behavior, in contrast to the asymmetric profile observed for PC-HT. This symmetry demonstrates that PC-FT is highly stable at planar conformations with a dihedral angle of 0° between the difluorothiophene and thiophene moieties, and the minimum energy is regained when the torsional angle completes a full 360° rotation. On the other hand, for PC-HT, the minimum energy conformation corresponds to a dihedral angle of −20°, which is in close agreement with the optimized value of 21.3° between the two moieties. The highly planar and symmetric structure observed for PC-FT is expected to propagate throughout the polymeric backbone and the thin film morphology, potentially enhancing the interchain interactions and charge transport properties.

Next, the energy of the highest molecular orbital (HOMO) was estimated to be −5.36, −5.78, −5.70, and −5.64 eV for PC12-HT, PC12-FT, PC16-FT, and PC20-FT, respectively, via Cyclic Voltammetry (CV) (Supporting Figure S3). The energy of the lowest unoccupied molecular orbital (LUMO) was calculated to be −3.69, −4.15, −4.07, and −4.01 eV for PC12-HT, PC12-FT, PC16-FT, and PC20-FT, respectively, based on the measured energy of HOMO and the optical band gaps (E_g_^opt^) using the most commonly used relation LUMO = HOMO + E_g_^opt^. The E_g_^opt^ values were 1.67 eV for PC12-HT and 1.63 eV for PC12-FT, PC16-FT, and PC20-FT (Figure 2c), as determined from the band edge of the Tauc plots for the thin films (Supporting Figure S4). Introduction of difluorothiophene into the SCP backbone contributes to lowering of the HOMO levels compared to the nonfluorinated analogues. This effect can be attributed to the stronger electron-withdrawing nature of the fluorine substituents. Prior studies have indicated a correlation between deeper HOMO levels and increased interchain hole mobility.^46,47^ Further, deeper HOMO levels help to increase the barrier for the hole injection in the off state, thereby suppressing the off-state current in organic FETs. The thermal stability (*T*_d_) of the SCPs was examined by differential scanning calorimetry (DSC) (Figure 2d) and thermogravimetric analysis (TGA) (Figure 2e). The onset temperatures of decomposition with 5% weight loss were 401.4 °C, 387.9 °C, 408.6 °C, and 409.6 °C for PC12-HT, PC12-FT, PC16-FT, and PC20-FT, respectively. Distinct melting temperatures (*T*_m_) at 213 °C, 236 °C, 166 °C, and 159 °C and crystallization temperatures (*T*_c_) at 197 °C, 208 °C, 149 °C, and 136 °C were observed for PC12-HT, PC12-FT, PC16-FT, and PC20-FT, indicating crystallization of the polymers. The high *T*_d_ values suggest a good thermal stability of the SCPs. Additionally, the slightly higher *T*_m_ for PC12-FT as compared to PC12-HT also confirms an improved crystal packing due to noncovalent S···F interactions. With increasing alkyl chain length in the PC-FT series, *T*_m_ and *T*_c_ systematically decreased, indicating that lower temperatures are required to anneal these SCPs.

### 2.2 Thin Film Characterization

#### Electronic Absorption Spectroscopy

**Figure 3:**
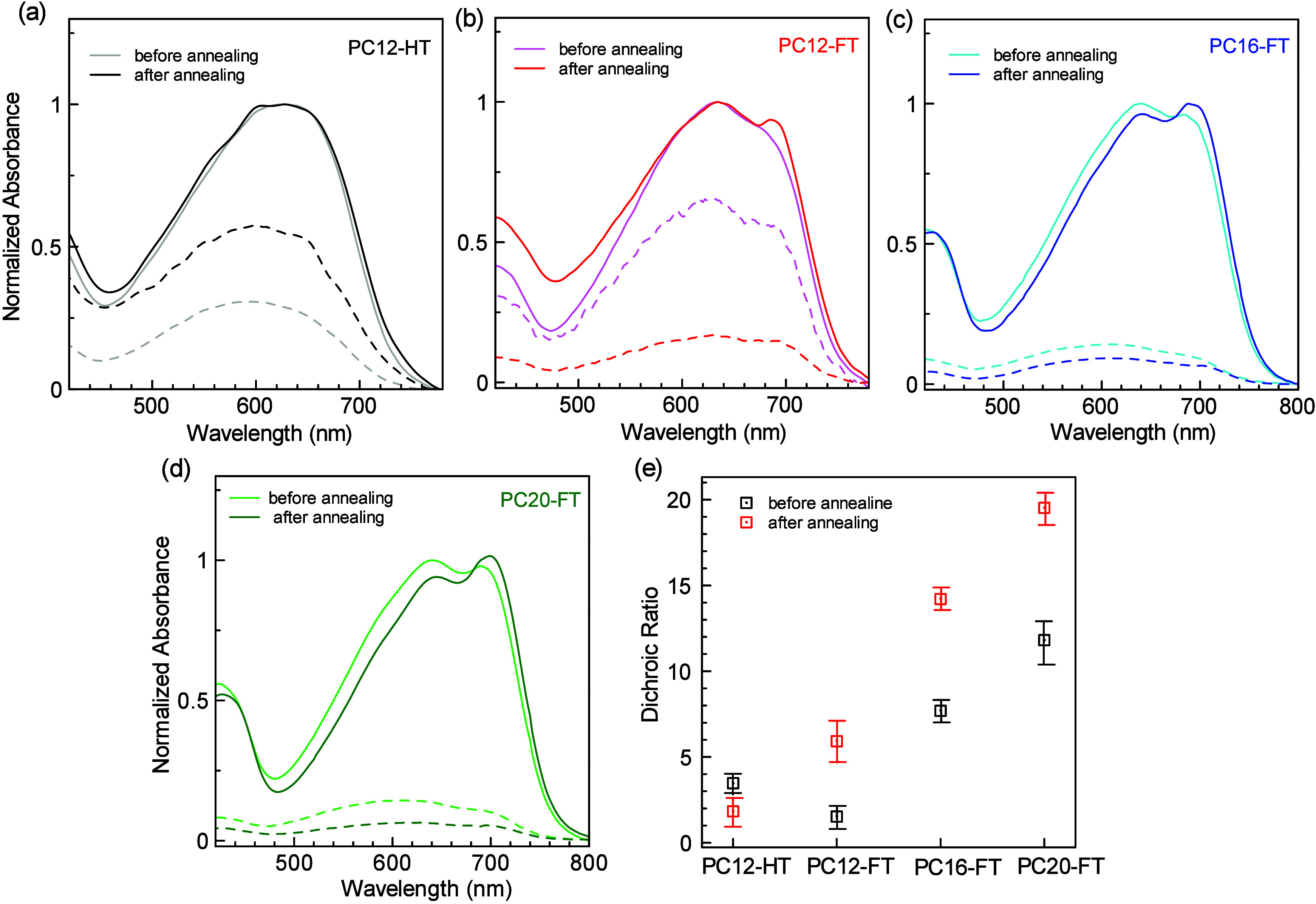
Electronic polarized absorption spectra of (a) PC12-HT, (b) PC12-FT, (c) PC16-FT, and (d) PC20-FT before and after annealing, normalized with respect to 0°. (solid = 0°; dotted = 90°). (e) Variation of dichroic ratio of all SCPs before and after annealing (averaged for 10 samples).

It has been reported by our group that UFTM thin films of SCPs are not only large in area and uniform but also exhibit uniaxial molecular orientation depending on the nature of SCPs as well as physical parameters such as concentration, temperature, and viscosity. Oriented thin films of UFTM fabricated PC12-HT, PC12-FT, PC16-FT, and PC20-FT and extent of the molecular orientation was estimated using polarized UV–visible-NIR absorption spectra placing a Glan-Thomson prism between the incident light source and sample as shown in Figure 3. The absorption peaks of crystalline π-conjugated SCP films are usually shifted to longer wavelengths (red shift) relative to those measured in solution (Supporting Figure S5). This behavior is due to the enhanced intermolecular interactions between the polymer chains and the π-conjugated SCP backbone, which enable the SCP chains to self-assemble in the solid state.^48,49^ The extent of the red-shift is related to the degree of molecular ordering in the SCPs. Here, a noticeable red-shift of 127, 110, 164, and 166 nm in the absorption spectra of PC12-HT, PC12-FT, PC16-FT and PC20-FT UFTM films, respectively, has been observed as compared to their solution state in chloroform. Further, the polarized absorption spectra of the SCP thin films were measured before and after annealing at their glass transition temperature (*T*_g_). The molecular orientation is estimated by the optical dichroic ratio (DR) before and after annealing the UFTM films. PC12-HT exhibited a DR of 3.47, which decreased to 1.83 after annealing (Figure 3a). On the other hand, PC12-FT showed a DR of 1.53, which increased to 5.91 after annealing at its respective *T*_g_ (Figure 3b). Fluorine-substituted SCPs with higher planarity show a higher degree of orientation due to stronger intermolecular interactions between the planar polymer chains. It can be explained considering the fact that planar chains can pack more tightly and orderly when assembled into thin films. The flat structure allows the polymer chains to align parallel and stack through π–π interactions. On the other hand, nonplanar structure of PC12-HT has a twisted backbone (Figure 2a) which may sterically hinder close packing of the chain, leading to low DR, which further decreased after annealing. The 0–0 transition peak pertaining to π–π stacking and 0–1 absorption peaks for PC12-FT was observed at 692 and 632 nm, respectively, which later exhibited a slight red shift of 2 nm after annealing. On the contrary, the PC12-HT only exhibited a 0–1 absorption peak at 626 nm, while the 0–0 transition peaks were missing. There is also an alternative mechanism to understand the appearance of 0–0 transition peaks and red-shift in the absorption spectra, namely J-aggregation. The Fluorine-substituted SCPs have a flatter molecular arrangement and an increased coupling between the transition dipoles, leading to an extended π-conjugation, thus resulting in pronounced J-aggregation.

Next, the molecular orientation of fluorine-substituted SCP thin films with varying alkyl chains was studied. It can be seen that with an increase in the alkyl chain length, the DR increases. The DR observed for PC12-FT, PC16-FT, and PC20-FT is 1.53, 7.69, and 11.81, respectively, before annealing and 5.91, 15.28, and 19.3, respectively, after annealing (Figure 3b,c,d). To the best of our knowledge, PC20FT with a DR of 19.3 demonstrated one of the highest orientations reported so far among the SCPs.^50−54^ We evaluated the DR of these SCP films across the wavelength spectrum and calculated the DR averaged across the wavelength spectrum (Supporting Figure S6). These average DR values are found to be 1.87, 6.72, 11.76, and 15.22 for annealed PC12HT, PC12FT, PC16FT, and PC20FT, respectively. These values are lower than the DR of the SCP thin films primarily due to the variation in absorption across the wavelength spectrum. Regions of high absorption result in higher DR, while regions of lower absorption lead to lower DR, thus reducing the overall average DR across the entire spectrum. Additionally, we obtained polarized absorption spectra with the polymer chains oriented at 45° angles relative to the polarizer. These spectra were normalized with respect to the parallel orientation and are shown in Supporting Figure S7. The absorption intensity at 45° is observed to fall approximately midway between the intensities measured in the parallel and perpendicular orientations. This observation demonstrates a clear correlation between the polymer chain orientation and absorption dynamics, providing further evidence for the anisotropic nature of these films. This can be attributed to longer alkyl side chains promoting interdigitation or interlocking between polymer backbones in the thin films. This tighter packing aligns the backbones and improves the orientation. Longer alkyl chains can help lock polymer backbones into oriented or aligned conformations, which even pronounce after annealing. A slight red-shift in the 0–0 transition peak and 0–1 absorption peak of 6 nm between C12-FT and C16-FT and 4 nm between C16-FT and C20-FT has also been observed. Moreover, an enhanced 0–0 transition peak has been seen after annealing these SCPs at their respective *T*_g_. This shows that as these LC-SCPs reach close to their *T*_g_, polymer chains reorganize into crystalline domains. According to Spano’s model, the 0–0 transition peak correlates the electronic structure of the exciton bandwidth with the transition energy of the intermolecular coupling.^55,56^ Thus, improved 0–0 transition peaks after annealing can be ascribed to reduced inhomogeneous broadening of the π–π transition attributed to tightly locked oriented polymer chains.

#### Thin Film Microstructure

**Figure 4:**
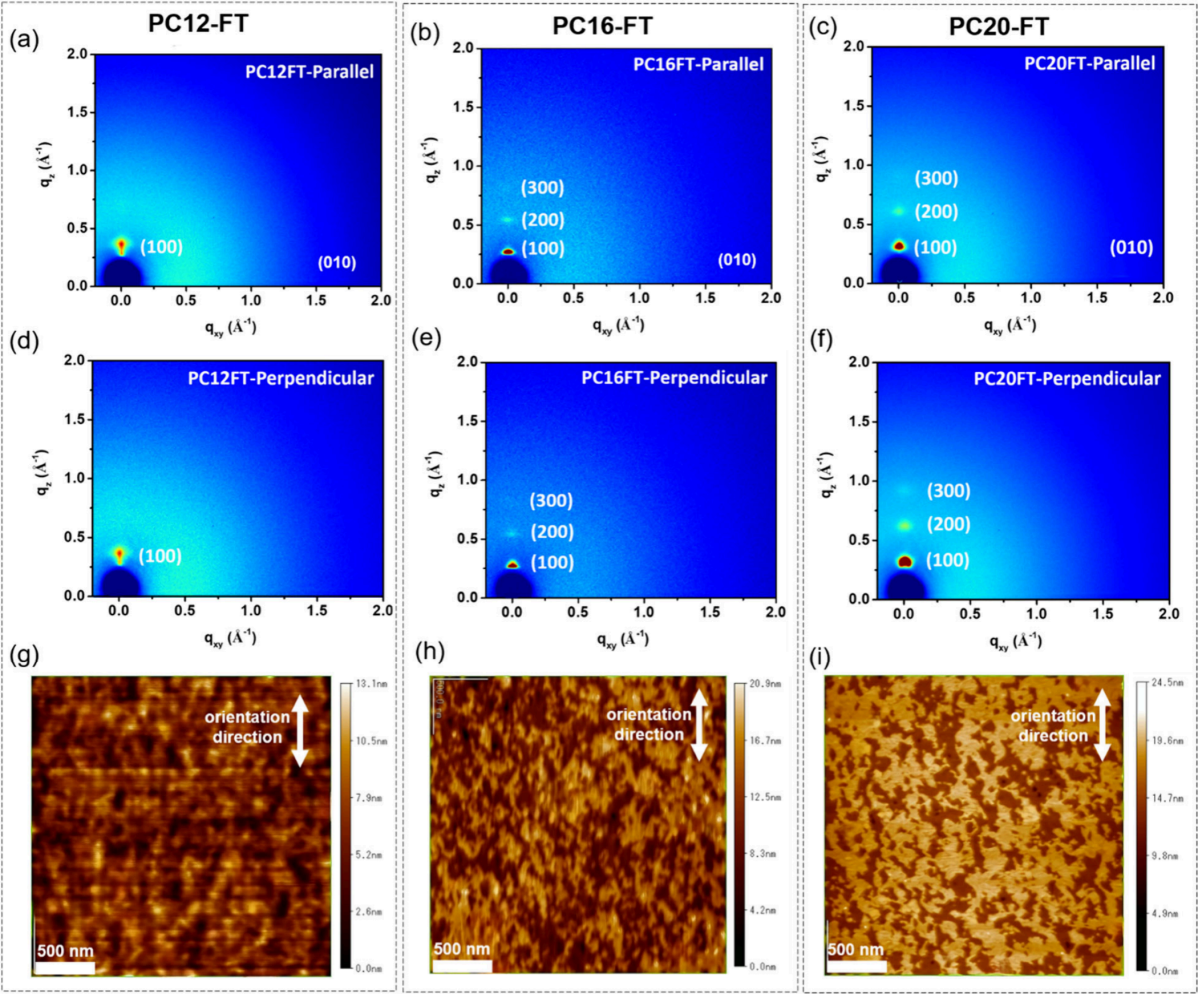
GIWAXS of PC12-FT in (a) parallel, (d) perpendicular direction, PC16-FT in (b) parallel, (e) perpendicular direction, and PC20-FT in (c) parallel, (f) perpendicular directions. AFM images of (g) PC12-FT, (h) PC16-FT, and (i) PC20-FT.

The thin films of SCPs under investigation coated with UFTM were subjected to thorough examination using 2D grazing incidence wide-angle X-ray scattering (GIWAXS) to elucidate the film crystallinity and molecular arrangements. Concurrently, atomic force microscopy (AFM) was also employed to complement the XRD findings and provide direct visualization of the surface morphology. Figure 4 displays the GIWAXS patterns and AFM images of the SCPs postannealing at their respective *T*_g_. Additionally, 2D graphs corresponding to all the SCPs are given in Supporting Figure S8. The GIWAXS profiles revealed pronounced reflection peaks of (h00), extending up to the first order for PC12-FT and up to the third order for PC16-FT and PC20-FT in the out-of-plane direction. Notably, the lamellar spacing corresponding to the alkyl stacking exhibited systematic increments with increasing alkyl side chain length, measuring 18.31 20.63, and 23.88 Å for PC12-FT, PC16-FT, and PC20-FT, respectively. The average crystallite size was estimated to be 17.4, 31.3, and 45.6 nm for PC12-FT, PC16-FT, and PC20-FT, respectively. Additionally, (010) peaks corresponding to π–π stacking are also present in the parallel direction and not visible in the perpendicular direction. This also shows that the thin films are anisotropic and edge-on oriented, thus facilitating the in-plane charge transport in FETs.^57,58^ Importantly, the intensity of the reflection peaks was found to increase with an increasing alkyl chain length, suggesting enhanced crystallinity. Subsequently, AFM analysis corroborated the GIWAXS findings, revealing distinct surface morphologies corresponding to different SCPs. Consistent with the GIWAXS results, longer alkyl chains exhibited larger domain sizes compared with shorter ones. The AFM images showcased well-defined domains with enhanced molecular ordering and size in PC20-FT thin films compared to its other derivative counterparts. The AFM image for PC12-HT oriented film has been shown in Supporting Figure S9. It can be seen that PC12-HT films have a lower degree of crystallinity and orientation as compared to PC12-FT, proving the importance of planarity in achieving higher orientation and crystallinity due to fluorine substitution in PC-FT films. Resulting from the findings of absorption spectroscopy, GIWAXS, and AFM, it is evident that longer alkyl chains present in a thin film geometry have greater configurational freedom to interdigitate between polymer backbones, enabling a tighter packing and ordering of the backbones. This enhanced order, propagated by the interdigitating alkyl chains, fosters an increased number of nucleation sequences, seeding the formation of crystalline domains during thin film fabrication and solidification. Consequently, the interdigitation and restricted geometry of longer alkyl chains serve as templates for improved backbone ordering, ultimately culminating in increased polymer crystallinity within the thin films.

### 2.3 Electrical Properties of Organic Field-Effect Transistors

**Figure 5:**
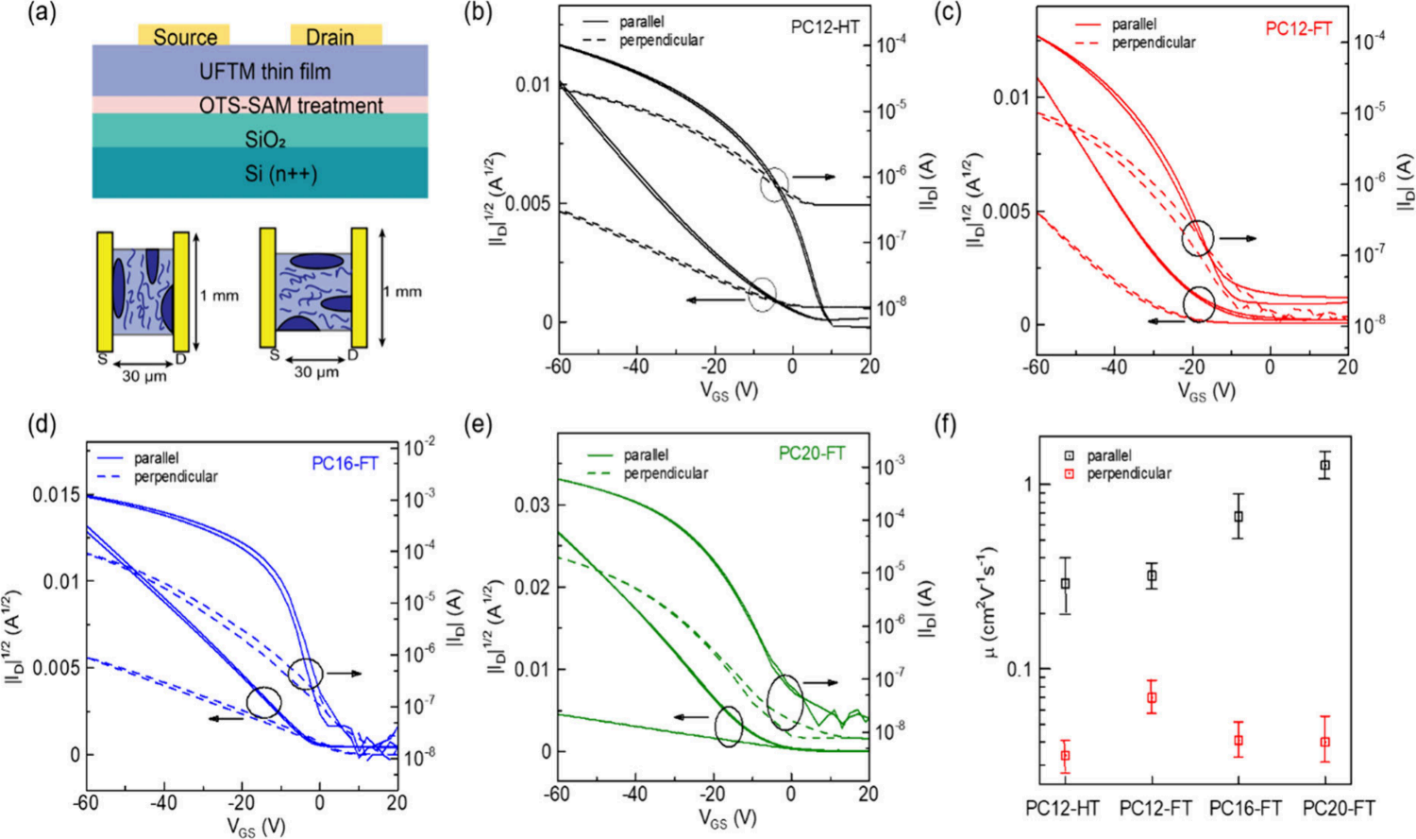
(a) Organic FET device architecture, transfer characteristic of (b) PC12-HT, (c) PC12-FT, (d) PC16-FT, and (e) PC20-FT in parallel and perpendicular directions. (f) Variation of μ for all SCPs in parallel and perpendicular directions (averaged for 20 devices).

The interesting disparities between nonfluorinated (PC12-HT) and fluorinated (PC-FT) substitutions, alongside variations in the alkyl chain length of PC-FTs, motivated an investigation into the electrical properties of thin films fabricated using these SCPs. To understand how the structural variations in planarity and crystallinity translate in to charge transport, bottom-gate top-contact FETs were fabricated in parallel and perpendicular to the channel direction between source and drain, following annealing of the thin films at their respective *T*_g_ (Figure 5a). For PC12-HT, the average field-effect mobility (μ) in the saturated regime was estimated to be 0.29 cm^2^V^–1^s^–1^ and 3.4 × 10^–2^ cm^2^V^–1^s^–1^ in parallel (μ^∥^_sat_) and perpendicular (μ^⊥^_sat_) directions to the channel, respectively (Figure 5b). This observation suggests enhanced carrier transport along the alignment direction of the polymer. The electrical anisotropy, represented by the ratio μ^⊥^_sat_/μ^∥^_sat_, was estimated to be 8.53. Conversely, for PC12-FT, the estimated μ^∥^_sat_ and μ^⊥^_sat_ values were 0.32 cm^2^V^–1^s^–1^ and 7.1 × 10^–2^ cm^2^V^–1^s^–1^, respectively, with an electrical anisotropy of 4.51 (Figure 5c). For PC16-FT, μ^∥^_sat_ and μ^⊥^_sat_ values were estimated to be 0.67 cm^2^V^–1^s^–1^ and 4.1 × 10^–2^ cm^2^V^–1^s^–1^, respectively, with an electrical anisotropy of 16.34 (Figure 5d). Finally, for PC20-FT μ^∥^_sat_ and μ^⊥^_sat_ values were estimated to be 1.27 cm^2^V^–1^s^–1^ and 4.01 × 10^–2^ cm^2^V^–1^s^–1^, respectively, with an electrical anisotropy of 31.67 (Figure 5e). The respective output characteristics are given in Supporting Figure S10. We also fabricated FETs using the four SCPs by keeping the orientation direction of the polymer chain 45° with respect to the channel direction as shown in Supporting Figure S11. The μ_*sat*_ for PC12-HT, PC12-FT, PC16-FT, and PC20-FT was determined to be 0.15 cm^2^V^–1^s^–1^, 0.19 cm^2^V^–1^s^–1^, 0.29 cm^2^V^–1^s^–1^, and 0.52 cm^2^V^–1^s^–1^, respectively. These values fall approximately between the μ^∥^_sat_ and μ^⊥^_sat_ values previously observed. These results corroborate the orientation-dependent charge transport behavior in the FETs, demonstrating a clear relationship between polymer chain alignment and device performance. Notably, fluorine-substituted SCPs (PC-FT), with their more planar backbone compared to their nonfluorinated counterparts, exhibited enhanced charge transport. Furthermore, there was a systematic increase in μ with an increase in alkyl chain length, consistent with our optical anisotropy and microstructural findings. It is essential to note that the effective conjugation lengths of SCPs are much smaller than the channel length, and charge transport is primarily governed by interchain hopping, occurring within a few nanometers at the semiconductor and gate dielectric interface. Large charge transport anisotropy in FETs prepared with PC20-FT indicates high in-plane alignment of SCPs in the films, which are in line with our optical anisotropic observations. The ratio of maximum current in the on- and off-state (I_on_/I_off_) in the best performing devices in parallel direction was ∼1 × 10^4^ - 7 × 10^4^ for the various FETs. It is crucial to accurately calculate FET mobility, as it is often overestimated due to nonlinear *I*_D_^1/2^ vs *V*_GS_ behavior, as reported in many high-mobility small molecule and polymer-based FETs.^59^ Notably, transfer characteristics of the FETs prepared with PC-FT with varying alkyl chain lengths in our case exhibited nearly ideal and linear *I*_D_^1/2^ vs *V*_GS_ characteristics with a nominal threshold voltage (*V*_th_) and saturated mobility (μ_sat_), with an exceptionally high-reliability factor (*r*) of 90–93%. Here, *r* < 100 and *r* > 100 indicate the degree of under- and overestimations, respectively [Supporting note 1].^59^

## 3 Conclusion

In this work, a series of donor–acceptor conjugated copolymers consisting of benzothiadiazole as an acceptor unit and thiophene-based donors without fluorine (PC12-HT) and with fluorine-substitution in the central thiophene ring of the donor unit (PC-FT) were strategically designed and synthesized to elucidate structure–property relationships. Incorporating electron-withdrawing fluorine atoms onto the thiophene unit enhanced backbone coplanarity through stabilized intrachain S···F interactions, which was confirmed by DFT calculations. This planarization contributed to deeper HOMO energy levels, bandgap reduction, and improved backbone ordering. Employing a range of materials characterization techniques (CV, DSC, and TGA), we revealed how molecular-level attributes translate to solid-state order and morphology. Increasing alkyl side chain length (PC12-FT, PC16-FT, PC20-FT) directly improved polymer solubility while promoting backbone alignment and tight chain packing owing to a higher degree of interdigitation. UFTM enabled the production of highly oriented semiconductor layers with exceptional control and consistency. This interplay manifested as progressively higher optical anisotropy with dichroic ratios of 5.9, 15.3, and 19.3 in PC12-FT, PC16-ft, and PC20-FT thin films. Correspondingly, GIWAXS and AFM analyses verified enhancement in lamellar order and crystallite size with longer alkyl chains. Finally, we interpreted these findings in the electrical applications by fabricating Organic FETs achieving a promising mobilities of 0.32, 0.67, and 1.24 cm^2^V^–1^s^–1^ for PC12-FT, PC16-FT, and PC20-FT and excellent reliability metrics. The trend in the electrical anisotropy with respect to the alkyl chain length was consistent with the optical and microstructural characterizations. This effective translation of molecular engineering into impressive charge transport properties highlights the promise of these SCPs for printed electronics.

## 4 Experimental Section

### 4.1 Materials

A novel conjugated polymer with molecular structures labeled as Poly[(3-(2-butyloctyl)-3′,4′-difluoro-5′-methyl-[2,2′-bithiophen]-5-yl)-7-(4-(2-butyloctyl)-5-methylthiophen-2-yl)benzo[c][1,2,5] thiadiazole] (PC12-HT), Poly[4-(3′,4′-difluoro-3-(2-hexyldecyl)-5′-methyl-[2,2′-bithiophen]-5-yl)-7-(4-(2-hexyldecyl)-5-methylthiophen-2-yl)benzo[c][1,2,5]thiadiazole] (PC12-FT), Poly[4-(3′,4′-difluoro-5′-methyl-3-(2-octyldodecyl)-[2,2′-bithiophen]-5-yl)-7-(5-methyl-4-(2-octyldodecyl)thioph-en-2-yl)benzo[c][1,2,5]thiadiazole] (PC16-FT), and Poly[4-(3-(2-butyloctyl)-5′-methyl-[2,2′-bithiophen]-5-yl)-7-(4-(2-butyloctyl)-5-methylthiophen-2-yl)benzo[c][1,2,5]thiadiazole] (PC20-FT) (as shown in Figure 1b) was successfully synthesized through a microwave-assisted and Palladium-catalyzed polycondensation reaction. Comprehensive information regarding the synthesis and characterization of these polymers is presented in Supporting Information. Gel-permeation chromatography was used to estimate the molecular weight of the synthesized polymer. The results of the molecular weight estimation, including the number-average molecular weight (*M*_n_), weight-average molecular weight (*M*_w_), and polydispersity index (PDI), are summarized in Table 1 above. Dehydrated chloroform, used as the solvent for the SCP ink, was purchased from Fujifilm Wako, Japan. For the subphase liquid, ethylene glycol (Eg) was purchased from Sigma-Aldrich. Octadecyl-trichlorosilane (OTS), used as the self-assembled monolayer for surface modification, was purchased from Sigma-Aldrich. Other solvents used for cleaning the substrates (acetone, methanol, and chloroform) were also purchased from Sigma-Aldrich.

**Table 1:** UFTM Film Fabrication Conditions

SCP	Concentration of SCP ink (mgml^-1^)	Temperature of subphase liquid (°C)
PC12-HT	30	50
PC12-FT	30	55
PC16-FT	50	50
PC20-FT	40	45

### 4.2 Thin Film Fabrication

SCP ink for the various polymers was prepared in dehydrated chloroform. The UFTM thin films were fabricated by optimizing various conditions to attain the best degree of orientation. In UFTM, there are primarily three conditions, concentration of the SCP ink, viscosity of the subphase liquid, and temperature of the subphase liquid. The optimized values for the various SCPs used in this work are given in Table 1, while the subphase liquid used for all four SCPs was ethylene glycol (viscosity = 6.24 cst.).

In UFTM, a drop (∼10 μL) of the SCP ink is dropped at the interface of a custom-made slider and the subphase liquid. Since the density of the SCP ink is less than the subphase liquid, the ink floats on to the subphase liquid. Due to the Marangoni effect, the film starts to spread, with gradual evaporation of the ink solvent, thus resulting in a solid thin film. Due to the viscosity imparted by the subphase liquid, the polymer domains are oriented perpendicular to the flow. This thin film is highly uniform and can be cast on any desired clean substrate. Later, the film fabricated on the substrate is washed using methanol to remove the excess liquid substrate and dried using a nitrogen blow. Later, the UFTM film was annealed at their respective *T*_g_. The *T*_g_ for PC12-HT, PC12-FT, PC16-FT, and PC20-FT was estimated to be 190 °C, 190 °C, 150 °C, and 120 °C, respectively.

### 4.3 SCP and Thin Film Characterization

#### DSC and TGA

The thermal properties of the samples were measured using a PerkinElmer DSC 8000 instrument through heating/cooling cycles from 50 to 250 °C at a heating rate of 10 °C min^–1^ in a nitrogen atmosphere. TGA was performed using a HITACHI STA 7200 thermogravimetric analyzer under a nitrogen flow. All samples were heated from 50 to 700 °C at a heating rate of 10 °C min^–1^.

#### Polarized Absorption Spectroscopy

The optical absorption spectra were recorded on a JASCO V-570 UV–visible-NIR spectrophotometer. An optical prism was used as a polarizer to analyze the orientation of thin films in the parallel and perpendicular direction. Prism was placed between glass substrate and source of incident light. Clean glass substrates were used to measure the absorption spectra of the thin films.

#### AFM

AFM images for the thin films of the SCPs were taken using Jeol SPM-5200 in contact mode. The orientation direction of the thin films was predetermined using a polarizer and hence used for the AFM microscopy. The obtained orientation direction in the AFM images is believed to be the same as that observed using the polarizer.

#### Electrochemical CV

Electrochemical CV was performed on an AUBOTECH SP-150 electrochemical analyzer with glassy carbon, Pt wire, and Ag/AgCl as working, counter, and reference electrodes, respectively, in 0.10 M Ferrocene/acetonitrile at a scan rate of 50 mV/s.

#### GIWAXS

The GIWAXS data were collected at beamline 17A of the National Synchrotron Radiation Research Center (NSRRC) in Taiwan, with a wavelength of 1.32 Å. Typical GIWAXS patterns were acquired at an incidence angle of 0.14°.

### 4.4 Device Fabrication

Bottom-gate top-contact Organic FETs were fabricated using heavily doped n-type silicon substrates (resistivity <0.005 Ω cm) with a 300 nm thermally grown SiO_2_ layer as the gate and gate dielectric. The Si/SiO2 substrates were cleaned by sonication in acetone, methanol, and chloroform in ultrasonication for 10 min before surface functionalization. The gate oxide layer was treated with a self-assembled monolayer (SAM) by immersing the substrates in a 20 mM solution of OTS in anhydrous chloroform for 36 h under inert atmosphere (C_i_ = 10 nFcm^–2^). Afterward, the functionalized substrates were sonicated in chloroform for 10 min to remove physiosorbed OTS molecules. The substrates were then thermally annealed at 100 °C in an argon glovebox prior to semiconductor deposition. Thin films of the UFTM small-molecule semiconductors were subsequently deposited by spin coating and annealed at their respective Tg. Finally, gold source and drain contacts (50 nm) were thermally evaporated under high vacuum (<10^–6^ Torr). The channel length and width were kept at 30 μm and 1 mm, respectively. The output and transfer characteristics of the fabricated Organic FETs were measured under a mild vacuum (∼10^–2^ Torr) using a Keithley 2612 source meter. Multiple devices were tested to obtain statistical transport parameters.

## Supporting Information Available

Detailed analysis of the novel polymers, thin film microstructures (Absorption, AFM, XRD) and device characterizations are reported (PDF)
