# Supplementary material for: Enhancing Optoelectronic Anisotropy in Highly Oriented Thin Films by Fluorine Substitution in Novel Semiconducting Polymers
Source: ACS Appl Mater Interfaces. 2024 Sep 17;16(38):51229–40. doi: 10.1021/acsami.4c08566 (PMC11440466; doi:10.1021/acsami.4c08566)
Supplement: Supplementary file 1 — am4c08566_si_001.pdf [file am4c08566_si_001.pdf]

Supporting Information

Enhancing optoelectronic anisotropy in highly oriented thin films by fluorine substitution in novel semiconducting polymers

Shubham Sharma[a†], Moulika Desu[a†], Guan-Lin Chen[b], Kai-Wei Tseng[b, c, d], Kumar Vivek Gaurav[a], Zhe-Yu Liu[b, c, e], Kuang-Hao Cheng[b, c, f], Safalmani Pradhan[a], Palraj Ranganathan[b, c], Pang-Hsiao Liu[b, c], Xiang-Ling Chiu[f], Hirofumi Tanaka[g], Jyh-Chien Chen[f], Chin-Ti Chen[h], Chi-An Dai[e], Leeyih Wang[b, c, d], and Shyam S. Pandey[a*]*

[a] Graduate School of Life Science and Systems Engineering, Kyushu Institute of Technology, 2-4 Hibikino, Wakamatsu, Kitakyushu, Fukuoka 808-0196, Japan

[b] Center for Condensed Matter Science, National Taiwan University, Taipei 10617, Taiwan.

[c] Center of Atomic Initiative for New Materials, National Taiwan University, Taipei 10617, Taiwan

[d] Institute of Polymer Science and Engineering, National Taiwan University, Taipei 10617, Taiwan.

[e] Department of Chemical Engineering, National Taiwan University, Taipei 10617, Taiwan.

[f] Department of Materials Science and Engineering, National Taiwan University of Science and Technology, Taipei, 10607, Taiwan.

[g] Department of Brain Science, Kyushu Institute of Technology, 2-4 Hibikino, Wakamatsu, Kitakyushu, Fukuoka 808-0196, Japan

[h] Institute of Chemistry, Academia Sinica, Taipei 11529, Taiwan

* Corresponding authors. E-mail: shyam@life.kyutech.ac.jp; leewang@ntu.edu.tw

† These authors contributed equally to this work.

Keywords: Semiconducting polymers; Thin films; Device Fabrication; Planarity; Organic field transistors; Fluorination.

Synthesis

All required reagents and solvents were commercially purchased and were used as received. Tributyl(4-(2-butyloctyl)thiophen-2-yl)stannane[1],tributyl(4-(2-hexyldecyl)thiophen-2-yl)stannane[1], tributyl(4-(2-octyldodecyl)thiophen-2-yl)stannane[1], 4,7-dibromobenzo[c][1,2,5]thiadiazole[2] and 2,5-bis(trimethylstannyl)thiophene[3] were prepared according to already reported literature procedures.

In this procedure, 4,7-dibromobenzo[c][1,2,5]thiadiazole undergoes microwave-assisted Stille coupling with three distinct thiophene derivatives: 4,7-bis(4-(2-octyldodecyl)thiophen-2-yl)benzo[c][1,2,5]thiadiazole, 4,7-bis(4-(2-hexyldecyl)thiophen-2-yl)benzo[c][1,2,5]thiadiazole, and 4,7-bis(4-(2-butyloctyl)thiophen-2-yl)benzo[c][1,2,5]thiadiazole, respectively. Pd(PPh3)4 is utilized as a catalyst to synthesize compound 1, with the side chains varying based on the specific thiophene employed. Subsequently, compound 1 undergoes bromination at room temperature for 12 hours in CHCl3 using N-bromosuccinimide (NBS) to produce monomer M1 with diverse side chains. The synthetic routes for monomers M2 are detailed in the supporting information.

Monomer synthesis

Synthesis of 4,7-bis(4-(2-butyloctyl)thiophen-2-yl)benzo[c][1,2,5]thiadiazole

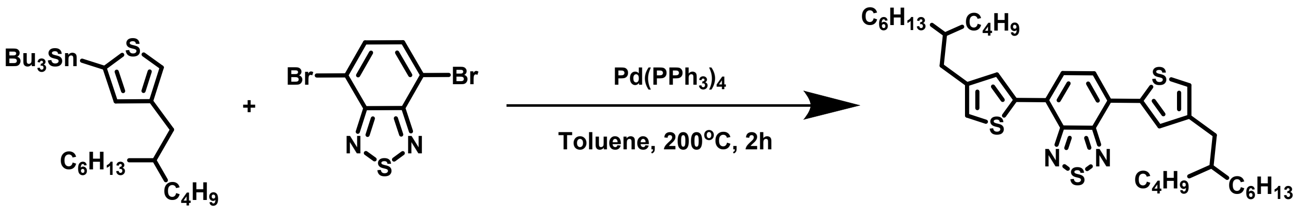

A reaction was performed using 6.0 g (0.011 mole) of Tributyl(4-(2-butyloctyl)thiophen-2-yl)stannane and 1.05 g (0.0036 mole) of 4,7-dibromobenzo[c][1,2,5]thiadiazole. The reaction took place in a microwave reaction vessel with the presence of $Pd(PPh_3)_4$ (10 mg) as the catalyst, and toluene (8 mL) as the solvent. The reaction mixture was heated to 200°C for 2 hours. Once the reaction was complete, the mixture was transferred to a 250 mL round-bottom flask, and the solvent was removed using rotary evaporation. The resulting product was then subjected to purification through column chromatography using hexane as the mobile phase. This process led to the formation of a red viscous gel (3.54 g, 50.2% yield). ^{1}H NMR (400 MHz, $CDCl_3$): δ 7.97-7.96 (d, J = 1.2 Hz, 2H), 7.85 (s, 2H), 7.03 (s, 2H), 2.66-2.64 (d, J = 6.8 Hz, 4H), 1.74-1.73 (m, 2H), 1.34-1.30 (m, 32H), 0.94-0.90

(m, 12H). ^{13}C NMR (100 MHz, $CDCl_3$): δ 152.5, 142.9, 138.7, 129.4, 125.8, 125.3, 122.3, 38.7, 34.9, 33.2, 32.9, 31.8, 29.6, 28.8, 26.5, 23.0, 22.6, 14.1, 14.0.

Synthesis of 4,7-bis(4-(2-hexyldecyl)thiophen-2-yl)benzo[c][1,2,5]thiadiazole

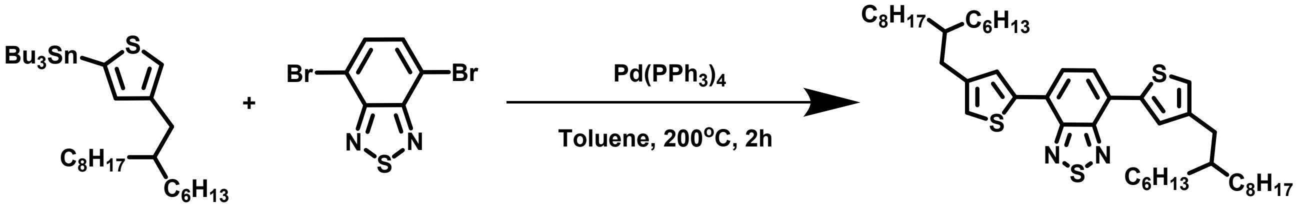

A reaction was performed using 4.38 g (0.0073 mole) of Tributyl(4-(2-hexyldecyl)thiophen-2-yl)stannane and 0.64 g (0.0022 mole) of 4,7-dibromobenzo[c][1,2,5]thiadiazole. The reaction took place in a microwave reaction vessel with the presence of $Pd(PPh_3)_4$ (10 mg) as the catalyst, and toluene (8 mL) as the solvent. The reaction mixture was heated to 200°C for 2 hours. Once the reaction was complete, the mixture was transferred to a 250 mL round-bottom flask, and the solvent was removed using rotary evaporation. The resulting product was then subjected to purification through column chromatography using hexane as the mobile phase. This process led to the formation of a red viscous gel (2.67 g, 48.8% yield). ^{1}H NMR (400 MHz, $CDCl_3$): δ 7.97-7.96 (d, J = 1.2 Hz, 2H), 7.85 (s, 2H), 7.03 (d, J = 1.2 Hz, 2H), 2.66-2.64 (d, J = 6.8 Hz, 4H), 1.73-1.72 (m, 2H), 1.33-1.28 (m, 48H), 0.91-0.87 (m, 12H). ^{13}C NMR (100 MHz, $CDCl_3$): δ 152.5, 142.9, 138.7, 129.4, 125.8, 125.3, 122.3, 38.8, 34.9, 33.2, 31.8, 29.9, 29.6, 29.5, 29.3, 26.5, 22.6, 14.0.

Synthesis of 4,7-bis(4-(2-octyldodecyl)thiophen-2-yl)benzo[c][1,2,5]thiadiazole

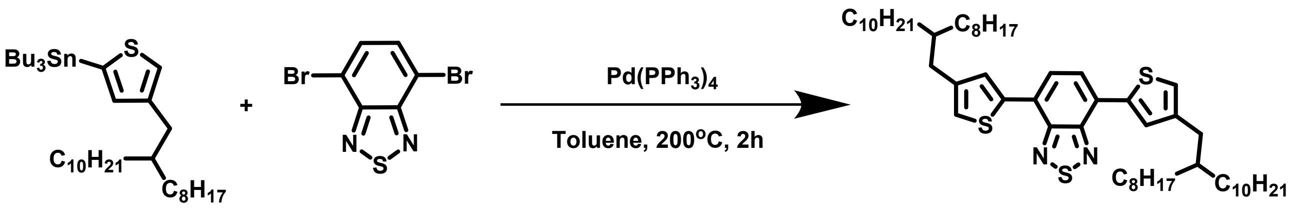

A reaction was performed using 2.94 g (0.0045 mole) of Tributyl(4-(2-octyldodecyl)thiophen-2-yl)stannane and 0.44 g (0.0015 mole) of 4,7-dibromobenzo[c][1,2,5]thiadiazole. The reaction took place in a microwave reaction vessel with the presence of $Pd(PPh_3)_4$ (10 mg) as the catalyst, and toluene (8 mL) as the solvent. The reaction mixture was heated to 200°C for 2 hours. Once the reaction was complete, the mixture was transferred to a 250 mL round-bottom flask, and the solvent was removed using rotary evaporation. The resulting product was then subjected to purification through column chromatography using hexane as the mobile phase. This process led to the formation of a red viscous gel (1.76 g, 45.6% yield). ^1H NMR (400 MHz, $CDCl_3$): δ 7.97-7.96 (d, J = 1.6 Hz, 2H), 7.85 (s, 2H), 7.03 (s, 2H), 2.66-2.64 (d, J = 6.8 Hz, 4H), 1.73-1.72 (d, 2H), 1.33-1.27 (m, 64H), 0.91-0.87 (m, 12H). ^{13}C NMR (100 MHz, $CDCl_3$): δ 152.5, 142.9, 138.7, 129.4, 125.8, 125.3, 122.3, 38.8, 35.0, 33.2, 31.8, 29.9, 29.6, 29.6, 29.3, 26.5, 22.6, 14.0.

Synthesis of 4,7-bis(5-bromo-4-(2-butyloctyl)thiophen-2-yl)benzo[c][1,2,5]thiadiazole

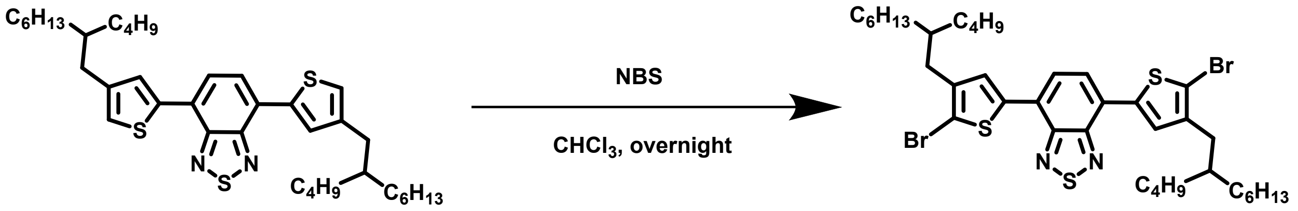

1.02 g (0.0016 mole) of 4,7-bis(4-(2-butyloctyl)thiophen-2-yl)benzo[c][1,2,5]thiadiazole was placed in a 100 mL round-bottom flask and mixed with 50 mL of chloroform ($CHCl_3$). The mixture was stirred until a uniform solution was obtained. Subsequently, N-Bromosuccinimide (NBS) (0.58 g, 0.0032 mole) was gradually introduced into the flask. The reaction vessel was sealed with a rubber stopper and allowed to proceed overnight. Upon completion of the reaction, it was terminated by adding deionized water. The resulting mixture was subjected to extraction using a combination of chloroform and deionized water. The organic layer was separated and dried using anhydrous magnesium sulfate to remove any water content. The solvent was then eliminated using rotary evaporation. The obtained product underwent purification via column chromatography, utilizing hexane as the mobile phase. This process yielded a red viscous gel (0.46 g, 60.2% yield). ^{1}H NMR (400 MHz, $CDCl_3$): δ 7.75-7.74 (d, J = 3.2 Hz, 2H), 2.60-2.58 (d, J = 1.2 Hz, 4H), 1.77 (s, 2H), 1.57-1.30 (m, 32H), 1.00-0.91 (m, 12H). ^{13}C NMR (100 MHz, $CDCl_3$): δ 152.1, 142.1, 138.1, 128.5, 125.2, 124.7, 112.1, 38.4, 34.1, 33.2, 32.9, 31.8, 29.6, 28.6, 26.4, 22.9, 22.6, 14.0.

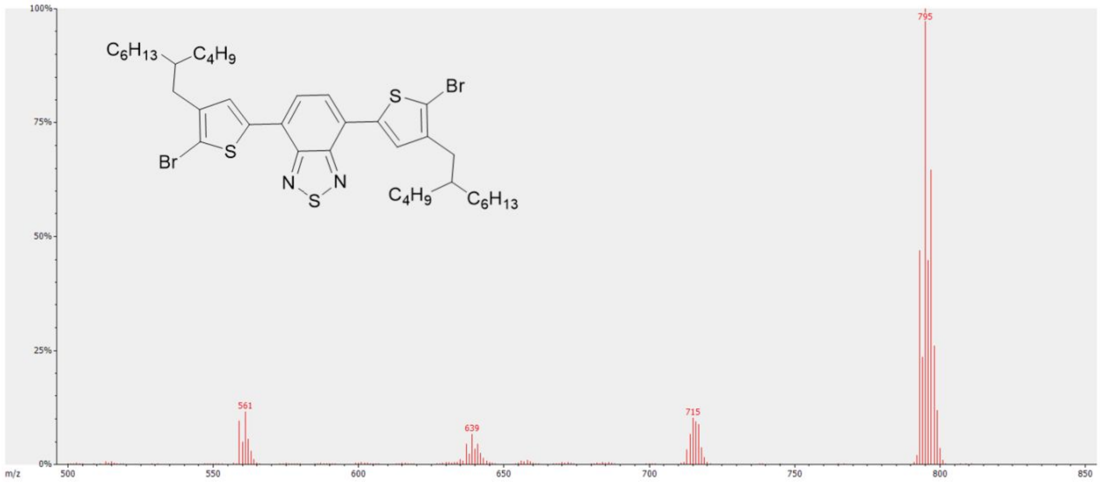

Synthesis of 4,7-bis(5-bromo-4-(2-hexyldecyl)thiophen-2-yl)benzo[c][1,2,5]thia-diazole

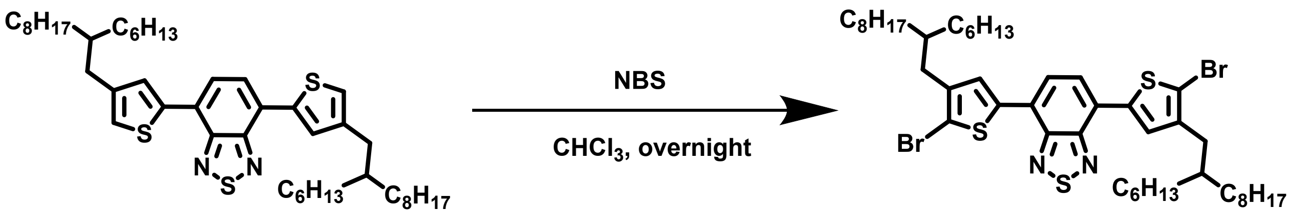

0.74 g (0.0010 mole) of 4,7-bis(4-(2-hexyldecyl)thiophen-2-yl)benzo[c][1,2,5]thiadiazole was placed in a 100 mL round-bottom flask and mixed with 50 mL of chloroform ($CHCl_3$). The mixture was stirred until a uniform solution was obtained. Subsequently, N-Bromosuccinimide (NBS) (0.35 g, 0.0020 mole) was gradually introduced into the flask. The reaction vessel was sealed with a rubber stopper and allowed to proceed overnight. Upon completion of the reaction, it was terminated by adding deionized water. The resulting mixture was subjected to extraction using a combination of chloroform and deionized water. The organic layer was separated and dried using anhydrous magnesium sulfate to remove any water content. The solvent was then eliminated using rotary evaporation. The obtained product underwent purification via column chromatography, utilizing hexane as the mobile phase. This process yielded a red viscous gel (0.63 g, 63.3% yield). ^{1}H NMR (400 MHz, $CDCl_3$): δ 7.78-7.76 (d, J = 1.6 Hz, 2H), 2.60-2.58 (d, J = 7.2 Hz, 4H), 1.77 (s, 2H), 1.34-1.28 (m, 48H), 0.89-0.86 (m, 12H). ^{13}C NMR (100 MHz, $CDCl_3$): δ 152.1, 142.1, 138.1, 128.5, 125.2, 124.7, 112.1, 38.4, 34.1, 33.2, 31.8, 29.9, 29.6, 29.5, 29.2, 26.4, 22.5, 14.0.

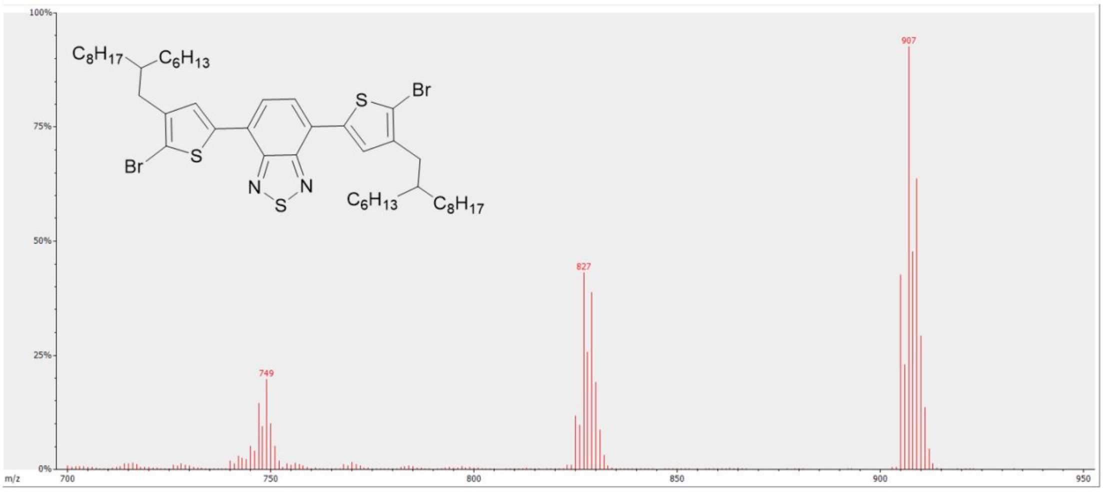

Synthesis of 4,7-bis(5-bromo-4-(2-octyldodecyl)thiophen-2-yl)benzo[c][1,2,5]thiadiazole

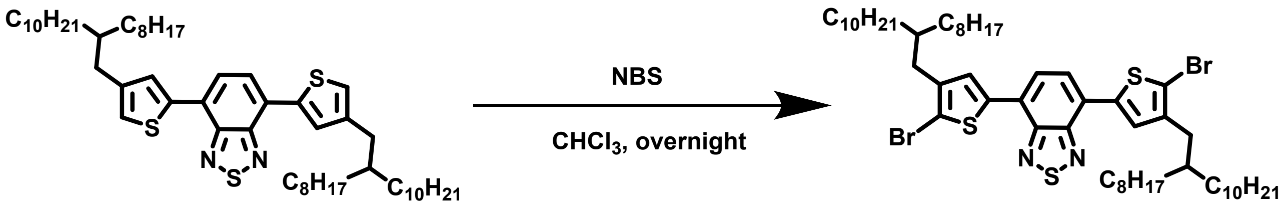

1.37 g (0.0016 mole) of 4,7-bis(4-(2-octyldodecyl)thiophen-2-yl)benzo[c][1,2,5]thiadiazole was placed in a 100 mL round-bottom flask and mixed with 50 mL of chloroform ($CHCl_3$). The mixture was stirred until a uniform solution was obtained. Subsequently, N-Bromosuccinimide (NBS) (0.57 g, 0.0032 mole) was gradually introduced into the flask. The reaction vessel was sealed with a rubber stopper and allowed to proceed overnight. Upon completion of the reaction, it was terminated by adding deionized water. The resulting mixture was subjected to extraction using a combination of chloroform and deionized water. The organic layer was separated and dried using anhydrous magnesium sulfate to remove any water content. The solvent was then eliminated using rotary evaporation. The obtained product underwent purification via column chromatography, utilizing hexane as the mobile phase. This process yielded a red viscous gel (0.93 g, 58.7% yield). ^{1}H NMR (400 MHz, $CDCl_3$): δ 7.77-7.76 (d, J = 4 Hz, 2H), 2.60-2.58 (d, J = 7.2 Hz, 4H), 1.77 (s, 2H), 1.58-1.26 (m, 64H), 0.88-0.86 (m, 12H). ^{13}C NMR (100 MHz, $CDCl_3$): δ 152.3, 142.2, 138.3, 128.7, 125.3, 124.8, 112.2, 38.5, 34.3, 33.4, 31.9, 30.0, 29.7, 29.6, 29.6, 29.3, 26.5, 22.6, 14.1.

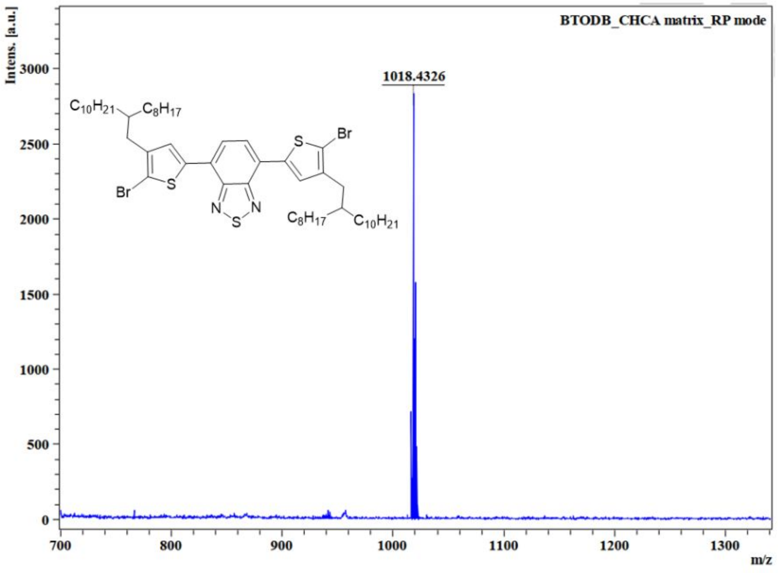

Synthesis of perbromothiophene

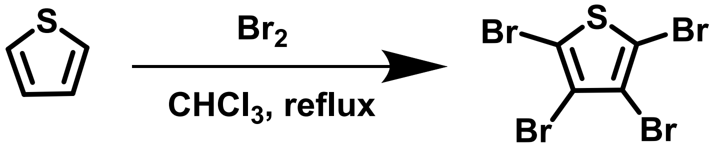

In a 500 mL two-neck flask, 18.0 g (0.21 mol) of furan was introduced, along with a magnetic stir bar and 150 mL of chloroform ($CHCl_3$). The setup included a reflux condenser attached to the upper neck and a 150 mL addition funnel attached to the side neck. Both of these openings were sealed with rubber septa. Through the addition funnel, bromine (64.74 mL, 1.26 mol) was gradually delivered using a syringe while the mixture was heated and kept at reflux conditions for a duration of 8 hours. Following the completion of the reaction, the mixture was cooled down in an ice bath and subsequently treated with a sodium hydroxide solution to neutralize the reaction. Extraction of the reaction mixture was carried out using a combination of $CHCl_3$ and deionized water. The organic layer was then dried using anhydrous magnesium sulfate. The solvent was eliminated utilizing a rotary evaporator, leading to the formation of a pale-yellow liquid, which was the crude product. The crude product was subject to purification by first washing it with a mixture of deionized water and ethanol in a 5:1 ratio. Further purification was accomplished by recrystallizing the product using $CHCl_3$ as the solvent. This process resulted in the production of a white solid (81.50 g, 97.1% yield). ^{13}C NMR (100 MHz, $CDCl_3$): δ 116.8, 110.2.

Synthesis of (3,4-dibromothiophene-2,5-diyl)bis(trimethylsilane)

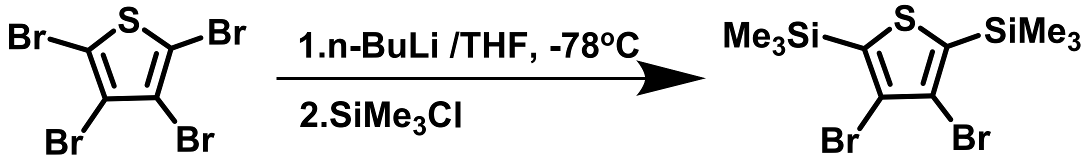

Using a 500 mL single-neck flask, 16.4 g (41.02 mmol) of perbromothiophene was placed, and a magnetic stir bar was included. The flask's neck was connected to a 100 mL dropping funnel, which was sealed with a rubber septum. To establish a low-moisture and low-oxygen environment, the reaction setup was evacuated and flushed with nitrogen gas on three occasions. Subsequently, 250 mL of dry tetrahydrofuran (THF) was introduced, and a solution of 2.5 M n-BuLi (in hexane) (37.6 mL, 0.094 mol) was carefully added dropwise to the dropping funnel. The reaction mixture was then cooled in a low-temperature bath, and the temperature was maintained at -78°C. Over a period of 2 hours, the n-BuLi solution was added dropwise to the flask, while the reaction continued to stir. Following the gradual addition of n-BuLi, the reaction mixture was stirred for an additional 10 minutes at the low temperature, after which trimethylsilyl chloride (11.58 mL, 0.902 mol) was introduced. The reaction was stirred under these conditions for the entirety of the night, allowing it to proceed at room temperature. Upon reaching completion, the reaction was terminated by adding deionized water. Extraction was performed using a combination of hexane and deionized water. The organic layer was isolated and subjected to drying with anhydrous magnesium sulfate. After concentrating the solution under reduced pressure, a pale-yellow liquid was obtained. The initial product underwent purification through column chromatography and was subsequently distilled, resulting in the formation of a clear liquid (11.54 g, 70.4% yield). ^{1}H NMR (400 MHz, $CDCl_3$): δ 0.45 (s, 18H). ^{13}C NMR (100 MHz, $CDCl_3$): δ 140.5, 122.1, -1.23.

Synthesis of (3,4-difluorothiophene-2,5-diyl)bis(trimethylsilane)

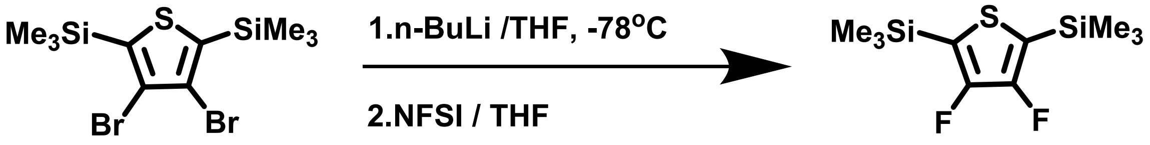

Introduce (3,4-dibromothiophene-2,5-diyl)bis(trimethylsilane) (15 g, 0.0375 mol) into a 500 mL dual-neck flask furnished with a magnetic stir bar. Attach a 100 mL addition funnel to the single neck of the flask and seal it using a rubber stopper. To establish a low-moisture and low-oxygen environment, evacuate the system and purge it with nitrogen gas three times. Inject 200 mL of dry tetrahydrofuran (THF) and introduce 2.5 M n-BuLi (in hexane) (34.17 mL, 0.084 mol) into the addition funnel. The flask, along with the magnetic stir bar, should be placed inside a low-temperature reactor, and the mixture should be uniformly stirred. Maintain the reaction temperature at -78°C. Begin dripping about half of the n-BuLi solution into the flask at a rate of approximately one drop per second. After completing the drip, allow the reaction to proceed for 1 hour. Next, dissolve N-Fluorobenzenesulfonimide (NFSI) (29.38 g, 0.093 mol) in 20 mL of anhydrous THF and add this solution to the addition funnel. Drip about half of this NFSI solution into the flask and stir the mixture at low temperature for 2 hours. Introduce the remaining portion of the n-BuLi solution and let the reaction continue for an additional 2 hours. Finally, add the remaining NFSI solution and stir the mixture at low temperature for 1 hour. Transition the flask to room temperature and allow it to stir overnight. After the reaction reaches completion, introduce deionized water to bring the reaction to a halt. Extract the resulting mixture with a combination of hexane and deionized water. Employ anhydrous magnesium sulfate to remove any water content and isolate the organic layer. Concentrate the organic layer using rotary evaporation, yielding a black gel-like substance. Purification of the crude product involves using a brief column chromatography process and subsequent distillation. These steps result in the acquisition of a clear liquid (7.41 g, 51.2% yield). ^{19}F NMR (376 MHz, $CDCl_3$): δ 131.4. ^{13}C NMR (100 MHz, $CDCl_3$): δ 152.3-149.5 (dd, *J* = 23.2, 259.7 Hz), 120.8-120.6 (dd, *J* = 7.7, 17.1 Hz).

Synthesis of 2,5-dibromo-3,4-difluorothiophene

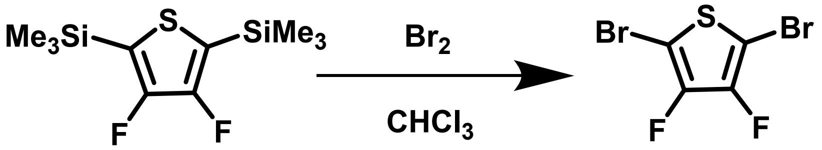

Place (3,4-difluorothiophene-2,5-diyl)bis(trimethylsilane) (16.8 g, 0.0624 mol) into a 250 mL round-bottom flask equipped with a magnetic stir bar, then introduce 50 mL of chloroform ($CHCl_3$). Seal the flask's opening with a rubber septum and, while in an ice bath, use a syringe to carefully add bromine (6.54 mL, 0.126 mol) to the reaction mixture. Allow the reaction to progress overnight at room temperature. Upon completion, introduce an aqueous sodium hydroxide (NaOH) solution to the reaction mixture while it's still in the ice bath to halt the reaction. Extract the resulting mixture using a combination of chloroform and deionized water. To eliminate any water content, introduce anhydrous magnesium sulfate to the organic layer. Employ rotary evaporation to concentrate the mixture, resulting in the formation of a crude brown liquid. Purification of the product entails column chromatography, where hexane is used as the mobile phase. Further purification is accomplished through low-temperature distillation. This process leads to the production of a transparent liquid (9.08 g, 52.4% yield). ^{19}F NMR (376 MHz, $CDCl_3$): δ 131.8. ^{13}C NMR (100 MHz, $CDCl_3$): δ 145.4-142.2 (dd, J = 20.0, 265.3 Hz), 90.3-90.1 (dd, J = 7.8, 16.0 Hz).

Synthesis of (3,4-difluorothiophene-2,5-diyl)bis(trimethylstannane)

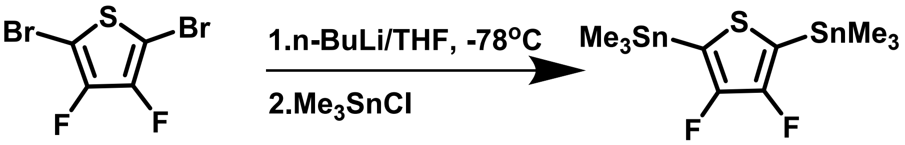

In a 250 mL single-neck flask, place 2,5-dibromo-3,4-difluorothiophene (2.23 g, 0.0080 mol) along with a magnetic stir bar. Connect a 100 mL addition funnel to the flask's upper opening and seal it using a serum stopper. Perform vacuum and nitrogen gas purging three times to establish a controlled environment with low moisture and oxygen content. Subsequently, introduce 150 mL of anhydrous tetrahydrofuran (THF). Into the addition funnel, introduce 2.5 M n-BuLi (in hexane) (7.06 mL, 0.0176 mol), and place the reaction system within a low-temperature reaction bath at -78°C. After thoroughly mixing the n-BuLi and THF, slowly drip the n-BuLi solution into the reaction flask at a rate of approximately 3 seconds per drop. Maintain the stirring of the reaction for 2 hours. Following this, incorporate trimethyltin chloride (20.09 mL, 0.0198 mol) into the flask and continue stirring the mixture at low temperature for 1 hour. Allow the reaction to continue stirring overnight at room temperature. Upon confirming the reaction's completion, conclude it by adding deionized water. Extract the mixture by employing a combination of hexane and deionized water. Use anhydrous magnesium sulfate to eliminate water from the organic layer. Concentrate the organic layer, resulting in the formation of a brown solid. For further refinement, subject the product to multiple recrystallization cycles using a mixture of methanol and hexane as solvents. Through this process, achieve the production of a white solid (1.62 g, 45.6% yield). ^{1}H NMR (400 MHz, $CDCl_3$): δ 0.41 (s, 18H). ^{19}F NMR (376 MHz, $CDCl_3$): δ -133.35. ^{13}C NMR (100 MHz, $CDCl_3$): δ 153.0-150.2 (dd, J = 25.3, 255.3 Hz), 119.9-119.5 (dd, J = 8.9, 44.2 Hz), -8.66.

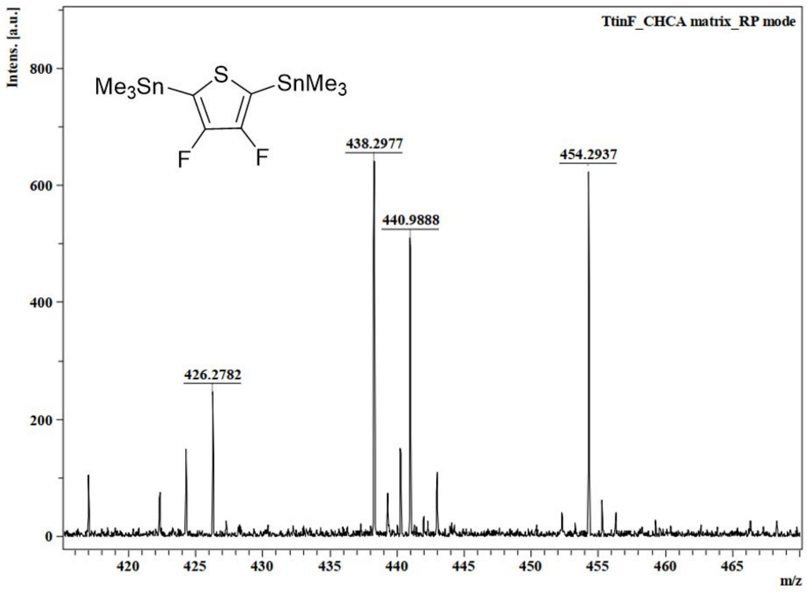
TtinF_CHCA matrix_RP mode
Intens. [a.u.]
Me3Sn
S
SnMe3
F
F
438.2977
454.2937
440.9888
426.2782
800
600
400
200
0
420
425
430
435
440
445
450
455
460
465
m/z

Polymer synthesis

Synthesis of PC12FT

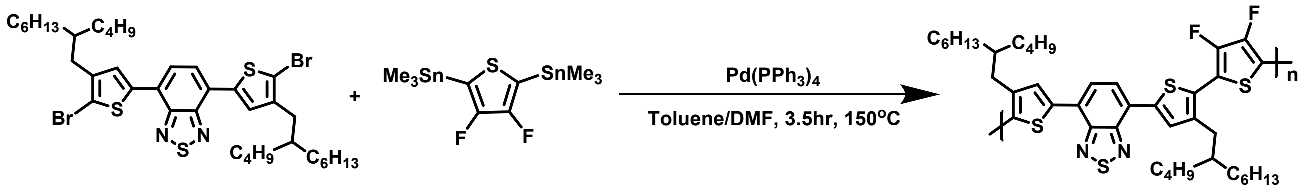

In a 10 ml microwave reaction vial, monomer 4,7-bis(5-bromo-4-(2-butyloctyl)thiophen-2-yl)benzo[c][1,2,5]thiadiazole (79.48 mg, 0.1 mmol) and monomer (3,4-difluorothiophene-2,5-diyl)bis(trimethylstannane) (44.57 mg, 0.1 mmol) were combined with $Pd(PPh_3)_4$ catalyst (12.27 mg, 0.0105 mmol). To this mixture, 4.6 ml of anhydrous toluene and 0.46 ml of DMF were added as the solvent. The concentration of the solution was 0.0197 M. The reaction mixture was sealed using a PTFE cap and placed within a microwave reactor, operating at 150˚C for a duration of 3.5 hours. Upon completion of the reaction, the resulting conjugated polymer was gradually dripped into methanol for reprecipitation. The solid product was then collected through filtration using a fritted funnel. The obtained conjugated polymer PC12FT underwent purification using a Soxhlet extractor, sequentially utilizing methanol, acetone, hexane, dichloromethane (DCM), and chloroform ($CHCl_3$). The final yield of the dark blue solid, identified as PC12FT, amounted to 74.8 mg, corresponding to a yield of 60.3%. ^{1}H NMR (400 MHz, $CDCl_3$): δ 8.05 (s, 2H), 7.92 (s, 2H), 2.80 (s, 4H), 1.82 (s, 2H), 1.34-1.28 (m, 32H), 0.91-0.87 (m, 12H). Elemental analyses calculated for [$C_{44}H_{60}F_2N_2S_4$]: C, 67.48%; H, 7.72%; N, 3.58%; S, 16.37%. Found: C, 66.73%; H, 7.26%; N, 3.57%; S, 16.11%. Molecular weight evaluated by GPC: M_n = 10.1 kDa, M_w = 15.8 kDa, PDI = 1.55.

Synthesis of PC16FT

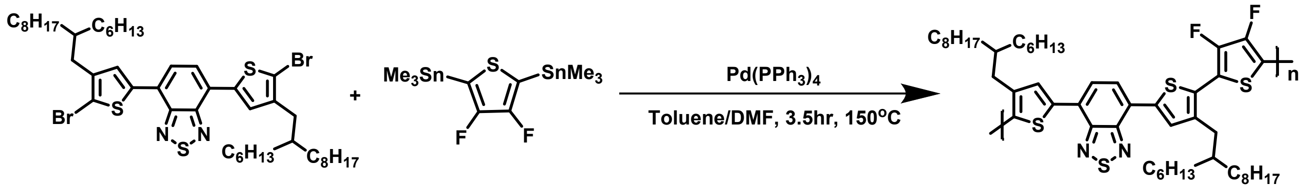

Monomer 4,7-bis(5-bromo-4-(2-hexyldecyl)thiophen-2-yl)benzo[c][1,2,5]thiadiazole (90.70 mg, 0.1 mmol) and monomer (3,4-difluorothiophene-2,5-diyl)bis(trimethylstannane) (44.57 mg, 0.1 mmol) were added with catalyst $Pd(PPh_3)_4$ (12.27 mg, 0.0105 mmol) to a 10 ml microwave reaction vial containing 4.6 ml of anhydrous toluene and 0.46 ml of DMF as the solvent. The concentration was 0.0197 M, and the reaction mixture was sealed with a PTFE cap and placed in a microwave reactor, set at 150˚C for 3.5 hours. After completion of the reaction, the conjugated polymer was slowly dripped into methanol for reprecipitation, followed by filtration using a fritted funnel to collect the solid product. The conjugated polymer PC16FT was purified using a Soxhlet extractor with methanol, acetone, hexane, DCM, and $CHCl_3$ in sequence. The final yield of the dark blue solid PC16FT was 82 mg, corresponding to a yield of 66.0%. ^{1}H NMR (400 MHz, $CDCl_3$): δ 8.05 (s, 2H), 7.92 (s, 2H), 2.80 (s, 4H), 1.83 (s, 2H), 1.34-1.28 (m, 48H), 0.89-0.84 (m, 12H). Elemental analyses calculated for [$C_{52}H_{78}F_2N_2S_4$]: C, 69.75%; H, 8.56%; N, 3.13%; S, 14.32%. Found: C, 69.29%; H, 8.15%; N, 3.11%; S, 13.95%. Molecular weight evaluated by GPC: M_n = 20.6 kDa, M_w = 30.1 kDa, PDI = 1.45.

Synthesis of PC20FT

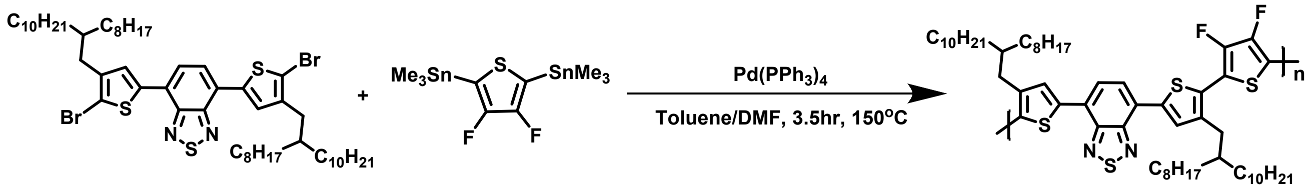

Monomer 4,7-bis(5-bromo-4-(2-octyldodecyl)thiophen-2-yl)benzo[c][1,2,5]thiadiazole (101.9 mg, 0.1 mmol) and monomer (3,4-difluorothiophene-2,5-diyl)bis(trimethylstannane) (44.57 mg, 0.1 mmol) were added with catalyst $Pd(PPh_3)_4$ (12.27 mg, 0.0105 mmol) to a 10 ml microwave reaction vial containing 4.6 ml of anhydrous toluene and 0.46 ml of DMF as the solvent. The concentration was 0.0197 M, and the reaction mixture was sealed with a PTFE cap and placed in a microwave reactor, set at 150˚C for 3.5 hours. After completion of the reaction, the conjugated polymer was slowly dripped into methanol for reprecipitation, followed by filtration using a fritted funnel to collect the solid product. The conjugated polymer PC20FT was purified using a Soxhlet extractor with methanol, acetone, hexane, DCM, and $CHCl_3$ in sequence. The final yield of the dark blue solid PC20FT was 107 mg, corresponding to a yield of 80%. ^{1}H NMR (400 MHz, $CDCl_3$): δ 8.05 (s, 2H), 7.91 (s, 2H), 2.79 (s, 4H), 1.82 (s, 2H), 1.34-1.24 (m, 64H), 0.88-0.85 (m, 12H). Elemental analyses calculated for [$C_{60}H_{92}F_2N_2S_4$]: C, 71.52%; H, 9.20%; N, 2.78%; S, 12.73%. Found: C, 70.45%; H, 8.67%; N, 2.86%; S, 12.68%. Molecular weight evaluated by GPC: M_n = 12.5 kDa, M_w = 22.9 kDa, PDI = 1.82.

Synthesis of PC12HT

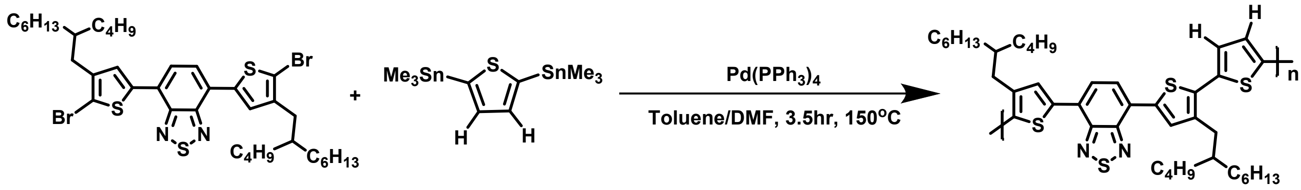

In a 10 ml microwave reaction vial, monomer 4,7-bis(5-bromo-4-(2-butyloctyl)thiophen-2-yl)benzo[c][1,2,5]thiadiazole (79.48 mg, 0.1 mmol) and monomer 2,5-bis(trimethylstannyl)thiophene (40.97 mg, 0.1 mmol) were combined with $Pd(PPh_3)_4$ catalyst (12.27 mg, 0.0105 mmol). To this mixture, 4.6 ml of anhydrous toluene and 0.46 ml of DMF were added as the solvent. The concentration of the solution was 0.0197 M. The reaction mixture was sealed using a PTFE cap and placed within a microwave reactor, operating at 150˚C for a duration of 3.5 hours. Upon completion of the reaction, the resulting conjugated polymer was gradually dripped into methanol for reprecipitation. The solid product was then collected through filtration using a fritted funnel. The obtained conjugated polymer PC12HT underwent purification using a Soxhlet extractor, sequentially utilizing methanol, acetone, hexane, dichloromethane (DCM), and chloroform ($CHCl_3$). The final yield of the dark blue solid, identified as PC12HT, amounted to 40.09 mg, corresponding to a yield of 75.3%. ^{1}H NMR (400 MHz, $CDCl_3$): δ 8.02 (s, 2H), 7.87 (s, 2H), 2.88 (s, 4H), 1.88 (s, 2H), 1.40-1.29 (m, 32H), 0.92-0.80 (m, 12H). Elemental analyses calculated for [$C_{44}H_{60}F_2N_2S_4$]: C, 70.73%; H, 8.36%; N, 3.75%; S, 17.6%. Found: C, 69.82%; H, 7.88%; N, 3.59%; S, 15.66%. Molecular weight evaluated by GPC: M_n = 15.6 kDa, M_w = 25.2 kDa, PDI = 1.61.

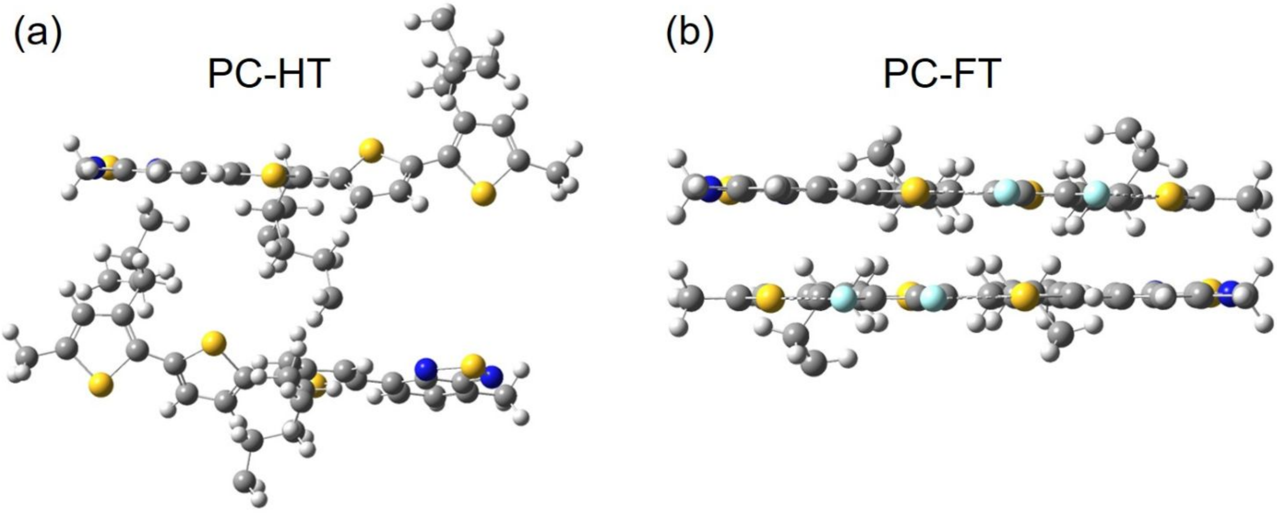

Figure S1: Cofacially placed monomer units of (a) PC-HT and (b) PC-FT in head-tail arrangement.

(a) <u>PC-HT</u>

Total energy of single monomer unit = -2616.4825 Hartree

Total energy of cofacially stacked monomer units = -5232.96674 Hartree

Binding energy = -0.00174 Hartree = -1.0629 kcal mol^{-1}

(b) <u>PC-FT</u>

Total energy of single monomer unit = -2814.8975 Hartree

Total energy of cofacially stacked monomer units = -5629.7991 Hartree

Binding energy = -0.00174 Hartree = -2.563 kcal mol^{-1}

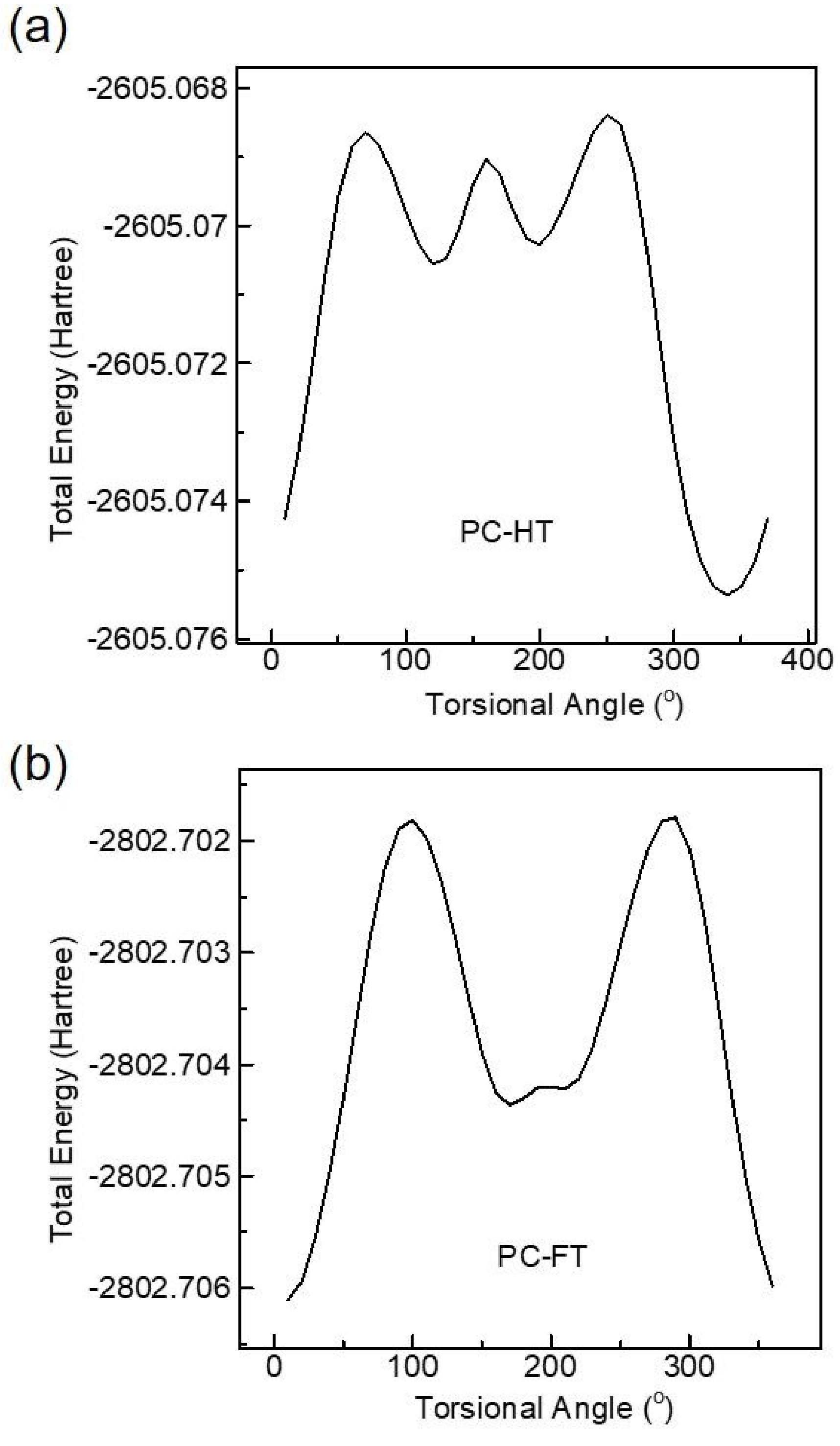

Figure S2: Torsional profiles of (a) PC-HT rotated around two thiophene moieties and (b) PC-FT rotated around difluorothiophene and thiophene moieties.

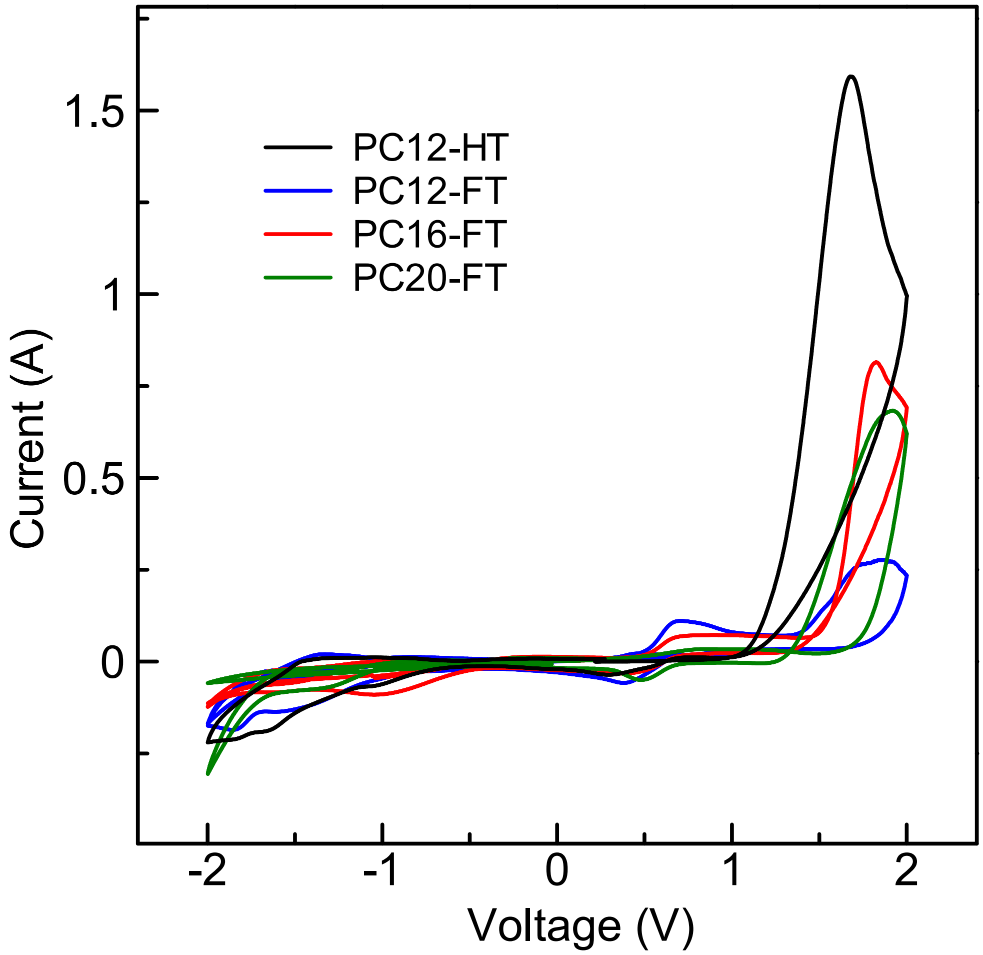

Figure S3: Current-Voltage characteristic showing Cyclic voltammetry for PC12-HT, PC12-FT, PC16-FT, and PC20-FT.

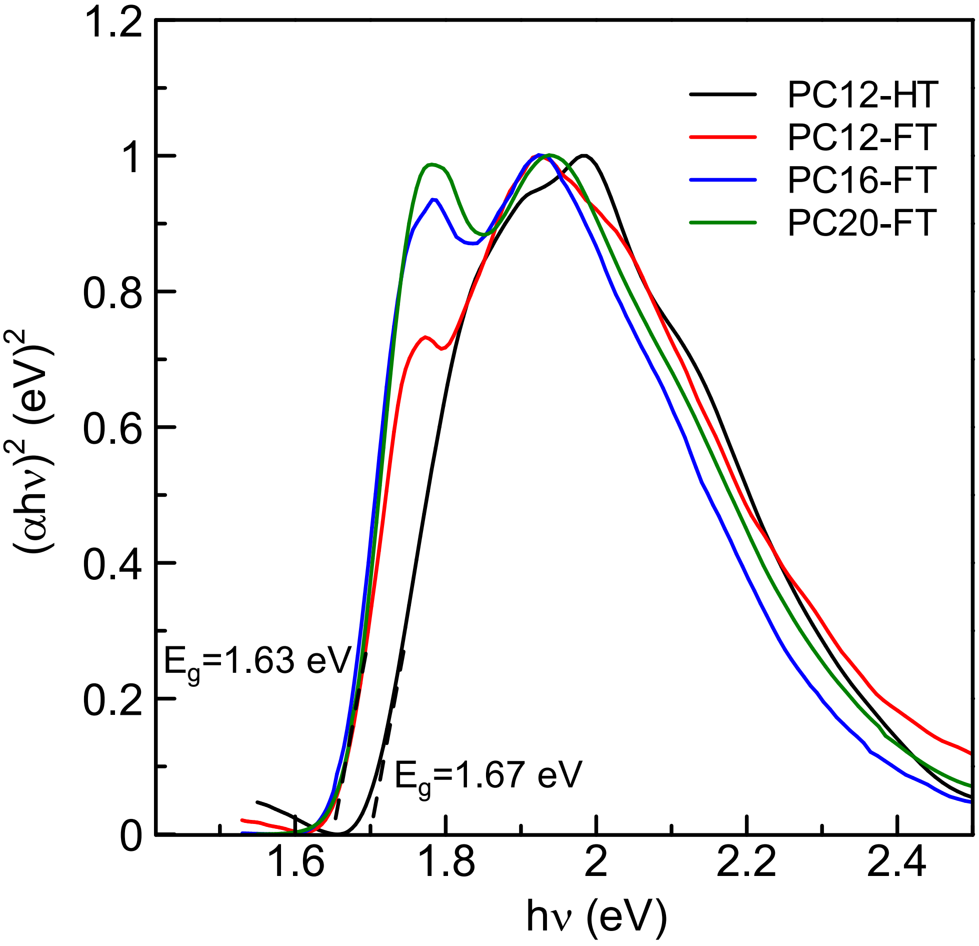

Figure S4: Tauc plot for PC12-HT, PC12-FT, PC16-FT, and PC20-FT UFTM thin films

The Tauc method is a well-established approach for determining the bandgap energy of a material by analyzing its absorption spectrum. In this method, the square of the absorption coefficient (α) multiplied by the photon energy (hν) is plotted against photon energy (hν). the Tauc plot exhibits a linear region at lower photon energies. Identify this linear portion of the plot, as it is essential for determining the bandgap. By extending the linear portion of the plot intersecting the x-axis gives an estimate of the bandgap energy (E_g). E_g for PC12-HT is found to be 1.67 eV, and 1.63 eV for PC12-FT, PC16-FT, PC20-FT.

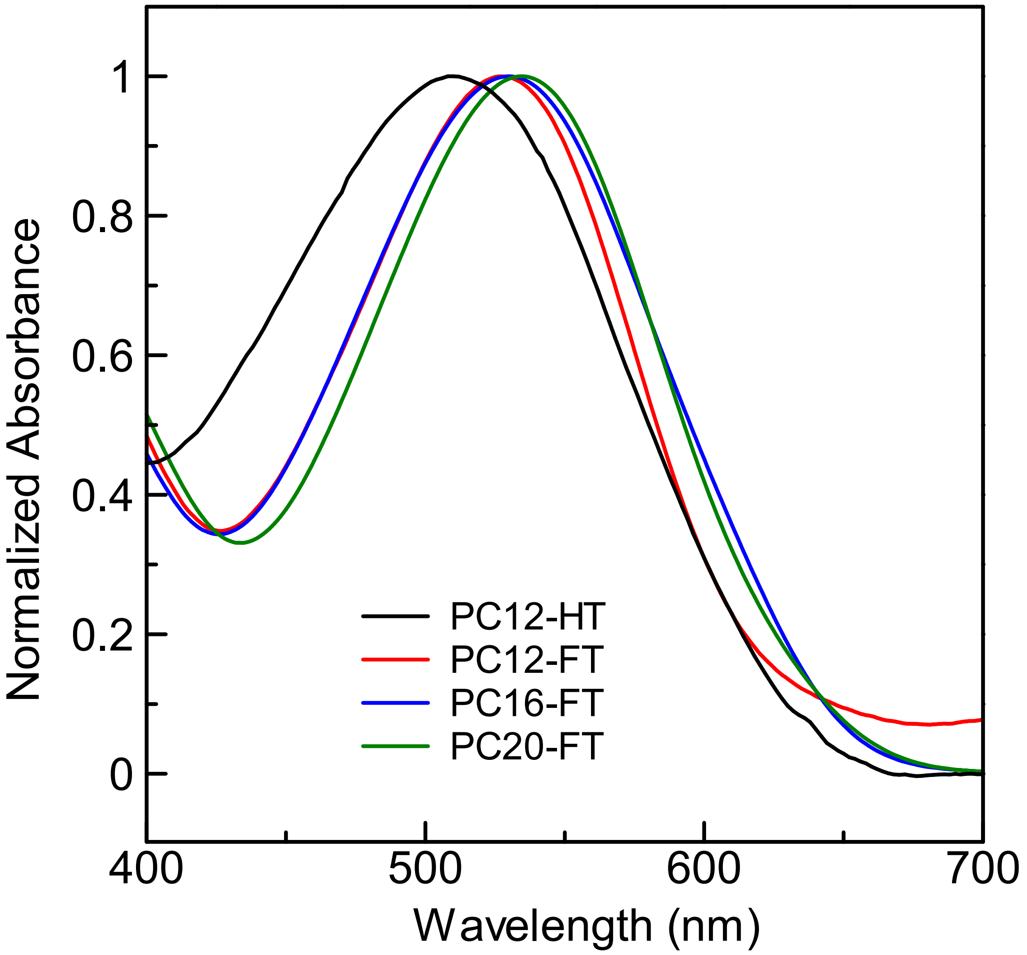

Figure S5: Solution state absorption spectra for PC12-HT, PC12-FT, PC16-FT, and PC20-FT.

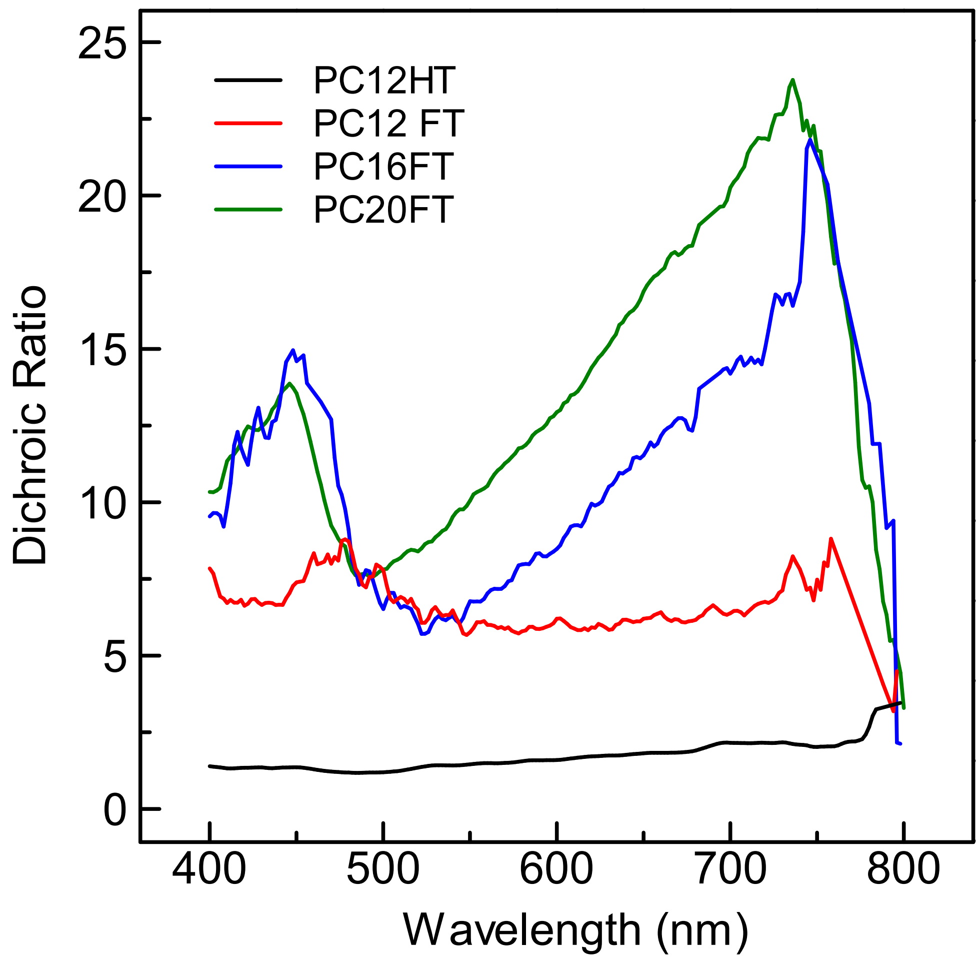

Figure S6: The Dichroic Ratio (DR) across the wavelength spectrum for PC12HT, PC12FT, PC16FT, and PC20FT. The DR averaged across the spectrum of the wavelength are 1.87, 6.72, 11.76, and 15.22, for PC12HT, PC12FT, PC16FT, and PC20FT, respectively.

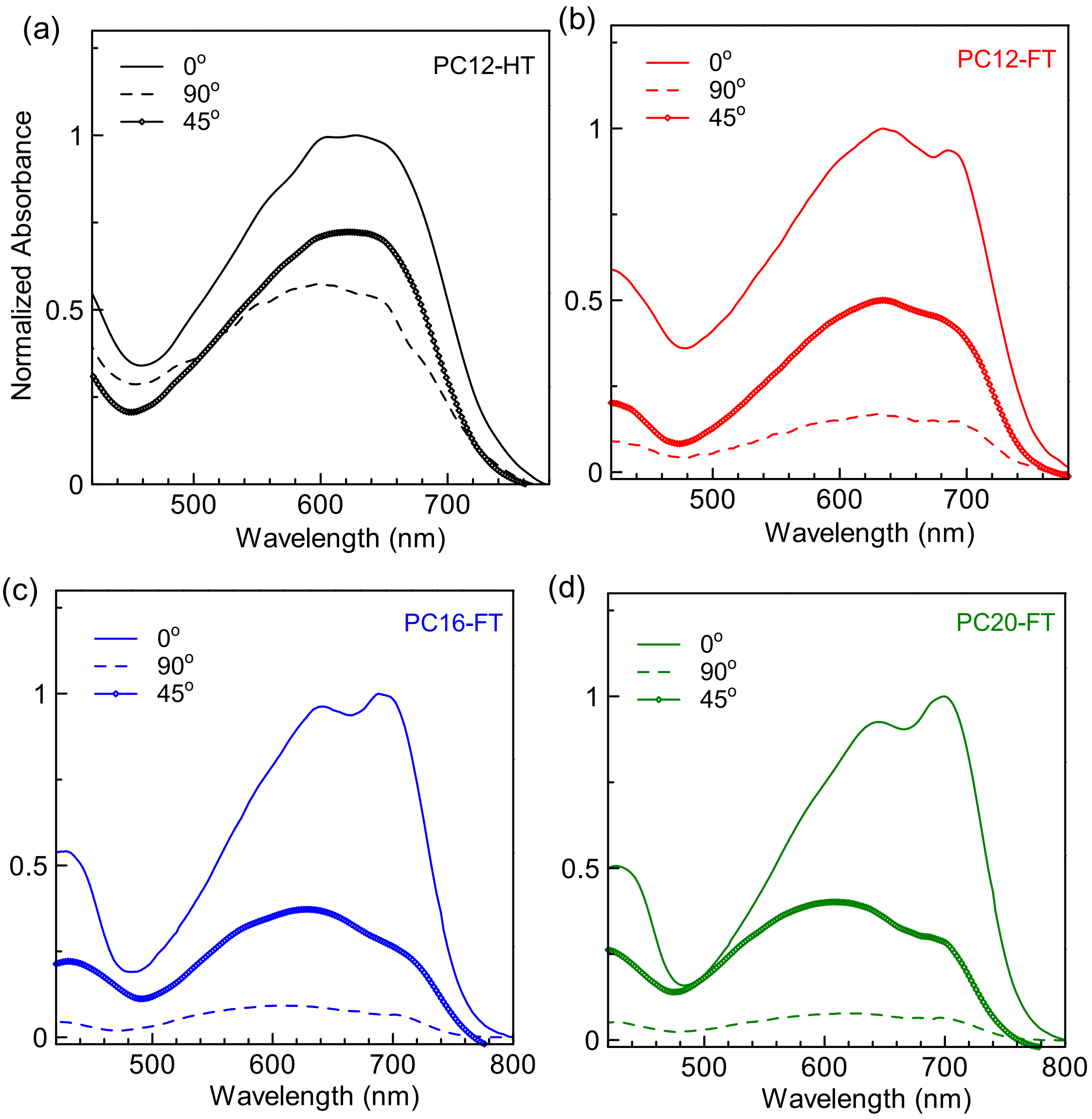

Supporting Figure S7: Normalized polarized absorption spectra for (a) PC12-HT, (b) PC12-FT, (c) PC16-FT, and (d) PC20-FT measured at 45° with respect to the polymer chain orientation.

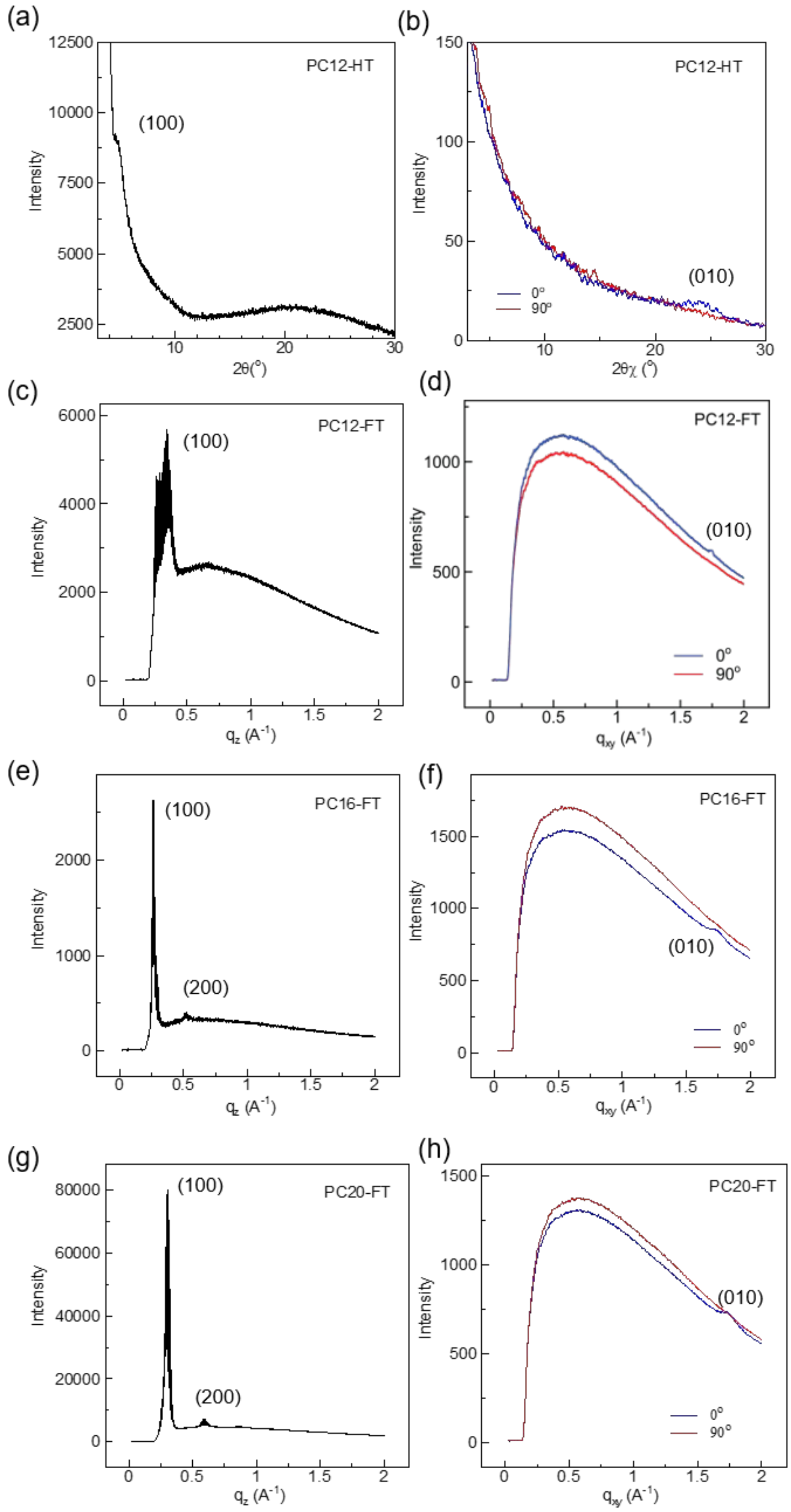

Figure S8: (a) Out-of-plane and (b) In-plane XRD of PC12-HT, (c) Out-of-plane and (d) in-plane XRD of PC12-FT, (e) Out-of-plane and (f) in-plane XRD of PC16-FT, and (g) Out-of-plane and (h) in-plane XRD of PC20-FT thin floating films.

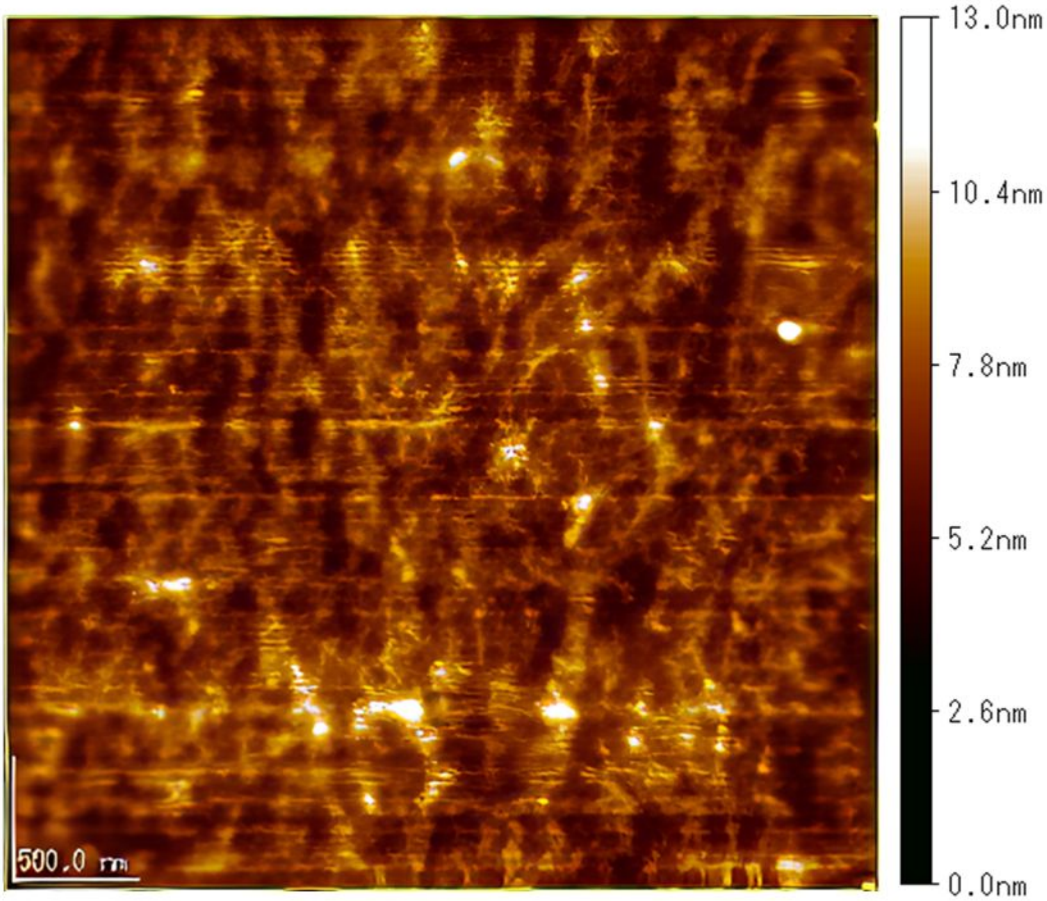

Figure S9: AFM image of PC12-HT floating thin films

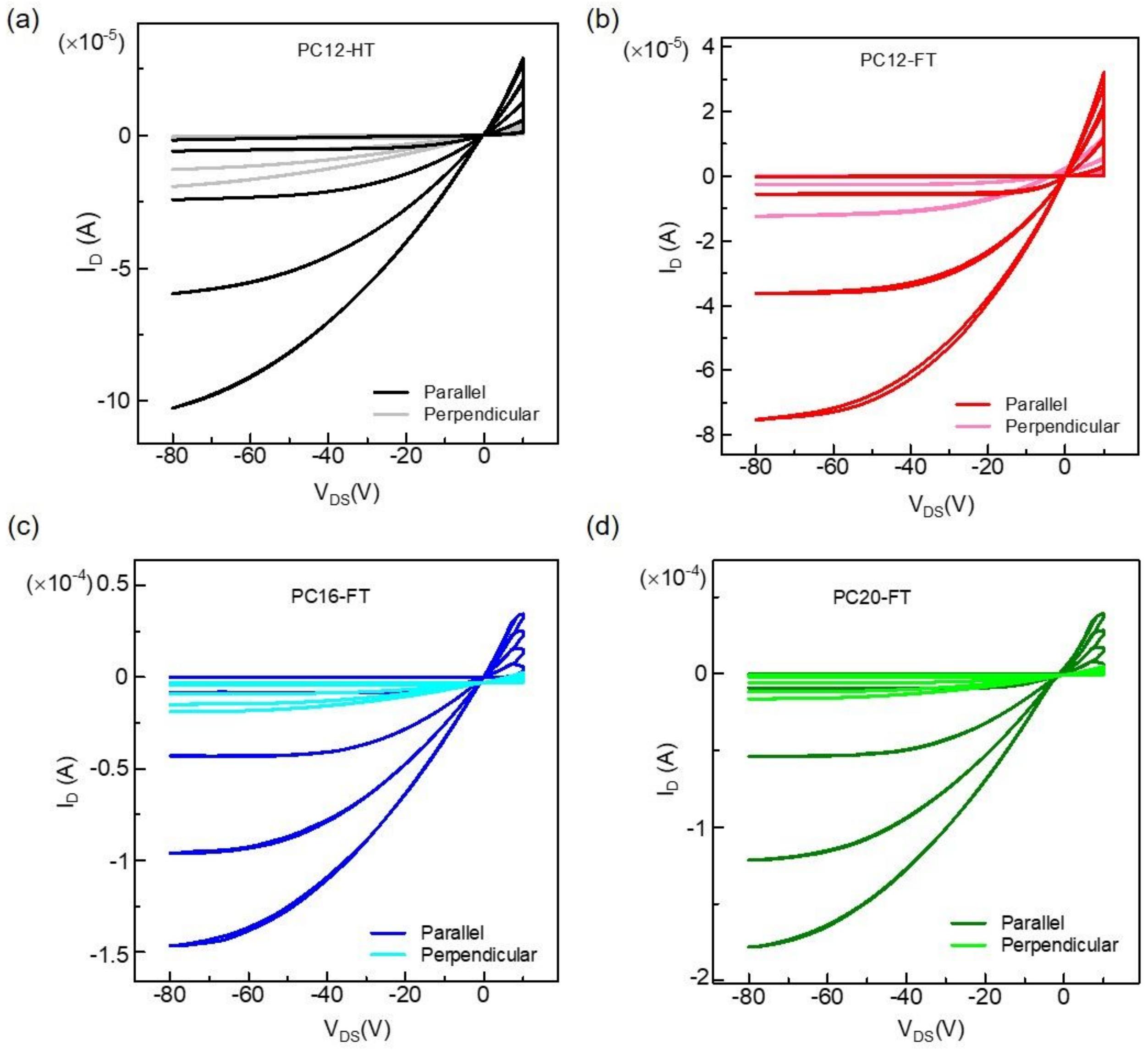

Figure S10: Output characteristics for (a) PC12-HT, (b) PC12-FT, (c) PC16-FT, and (d) PC20-FT with V_g varying from 0V to -60V.

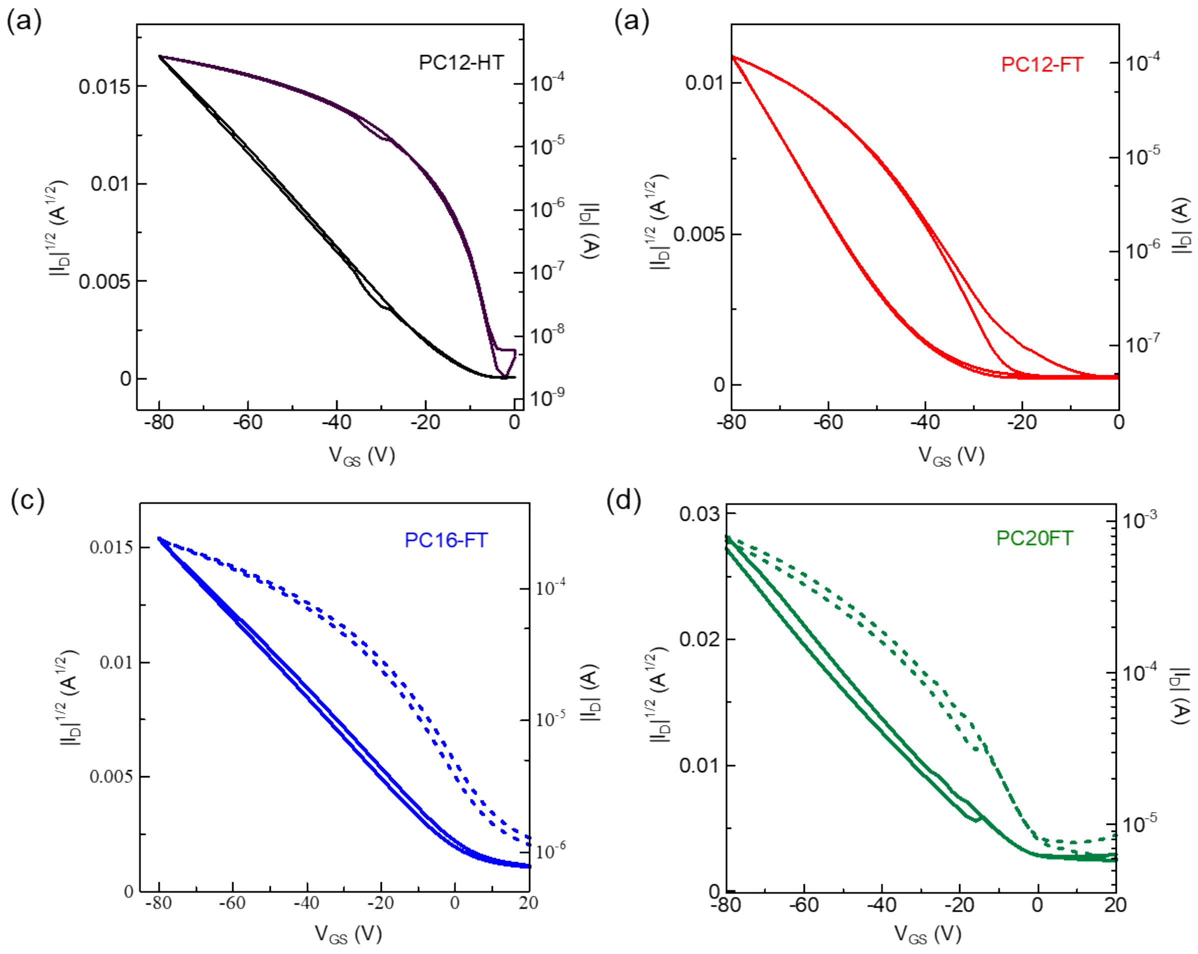

Supporting Figure S11: Transfer characteristics of OFETs fabricated by keeping the orientation direction of the polymer chain is 45° with respect to the channel direction. This experiment was conducted by rotating the position of the oriented thin film at 45° with respect to the channel direction during device fabrication. The saturated mobilities were recorded to be 0.15 $cm^2V^{-1}s^{-1}$, 0.19 $cm^2V^{-1}s^{-1}$, 0.287 $cm^2V^{-1}s^{-1}$, and 0.52 $cm^2V^{-1}s^{-1}$, respectively for (a) PC12-HT, (b) PC12-FT, (c) PC16-FT, and (d) PC20-FT.

Supporting Note 1:

In order to understand the misinterpretation of data, we also calculated the measurement reliability factor using the Eq. (i) and (ii) for saturated and linear regions.

$$r_{sat} = \frac{\left[\frac{\sqrt{|I_{SD}^{max}|} - \sqrt{|I_{SD}^{0}|}}{|V_{GS}^{max}|}\right]^2}{\left[\frac{WC_i}{2L}\mu_{sat}\right]} \qquad \text{Eq. (i)}$$

and

$$r_{lin} = \frac{\left[\frac{|I_{SD}^{max}| - |I_{SD}^{0}|}{|V_{GS}^{max}|}\right]}{\left[\frac{|V_{SD}|WC_i}{L}\mu_{lin}\right]} \qquad \text{Eq. (ii)}$$

where r_{sat} and r_{lin} are reliability factor in saturated and linear regimes, μ_{sat} and μ_{lin} are charge carrier mobility in saturated and linear regimes, $|I_{SD}^{max}|$ is the experimental maximum source-drain current reached at maximum gate voltage $|V_{GS}^{max}|$. $|I_{SD}^{0}|$ denotes the source-gate current at $V_{GS} = 0$.

Reference

[1] Nishinaga S, Mori H, Nishihara Y. Phenanthrodithiophene-isoindigo copolymers: effect of side chains on their molecular order and solar cell performance. *Macromolecules* **2015**, 48, 9, 2875–2885.

[2] DaSilveira Neto BA, Lopes ASA, Ebeling G, Gonçalves RS, Costa VEU, Quina FH, Dupont J. Photophysical and electrochemical properties of π-extended molecular 2,1,3-benzothiadiazoles. *Tetrahedron* **2005**, 61, 10975–10982.

[3] Liu Y, Zuo Y, Li S, Li J, Li L, Liu C, Ashraf S, Li P, Wang B. Synthesis and fine-tuning the pore properties of a thiophene based porous organic framework by post-oxidation treatment. *J. Mater. Chem. A*, **2019**,7, 21953–21958.
